# Supplementary figures and images for: Sex- and stage-dependent expression patterns of odorant-binding and chemosensory protein genes in Spodoptera exempta
Source: PeerJ. 2021 Sep 13;9:e12132. doi: 10.7717/peerj.12132 (PMC8445084; doi:10.7717/peerj.12132)

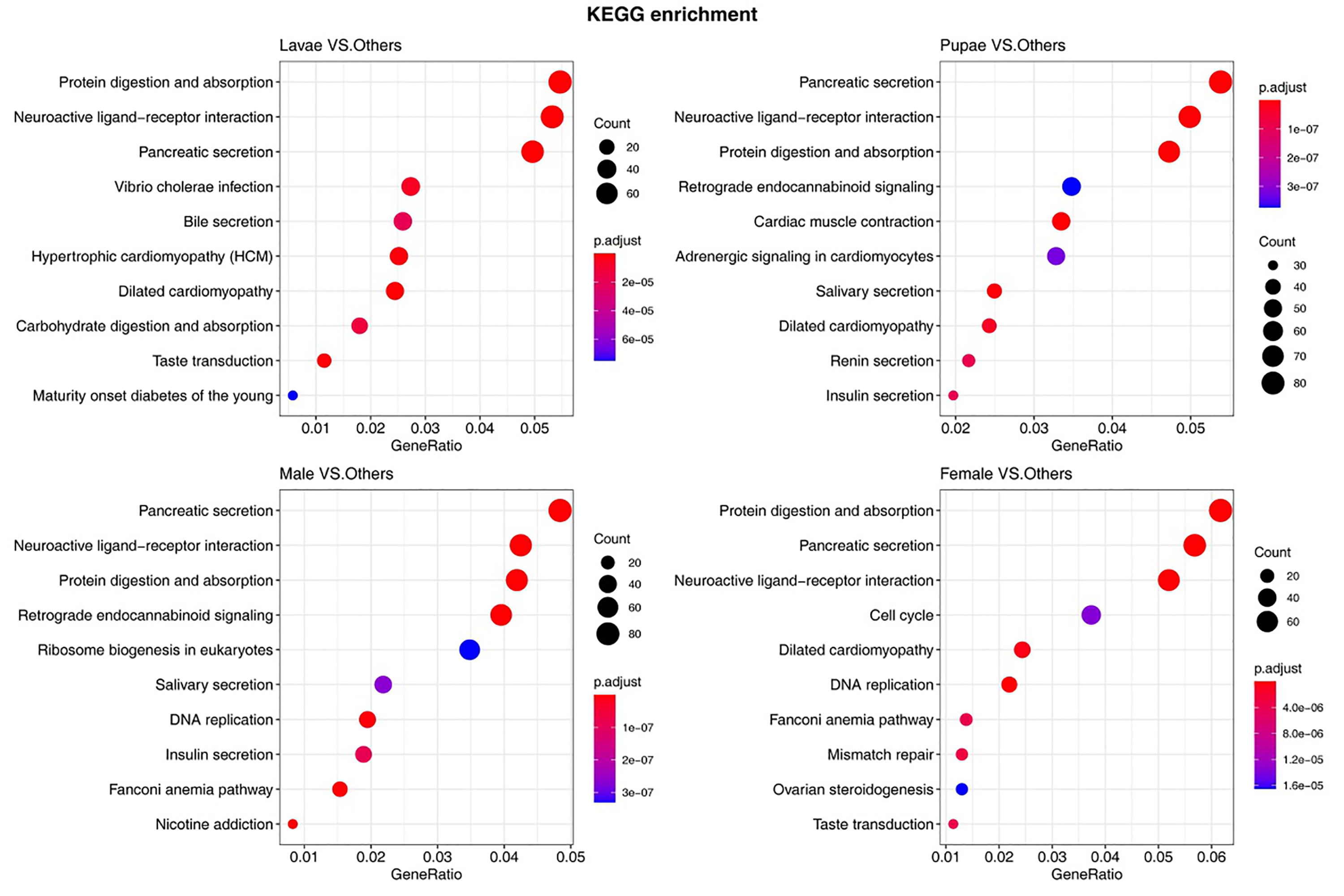

Supplement: Supplemental Information 1 [file peerj-09-12132-s001.png]

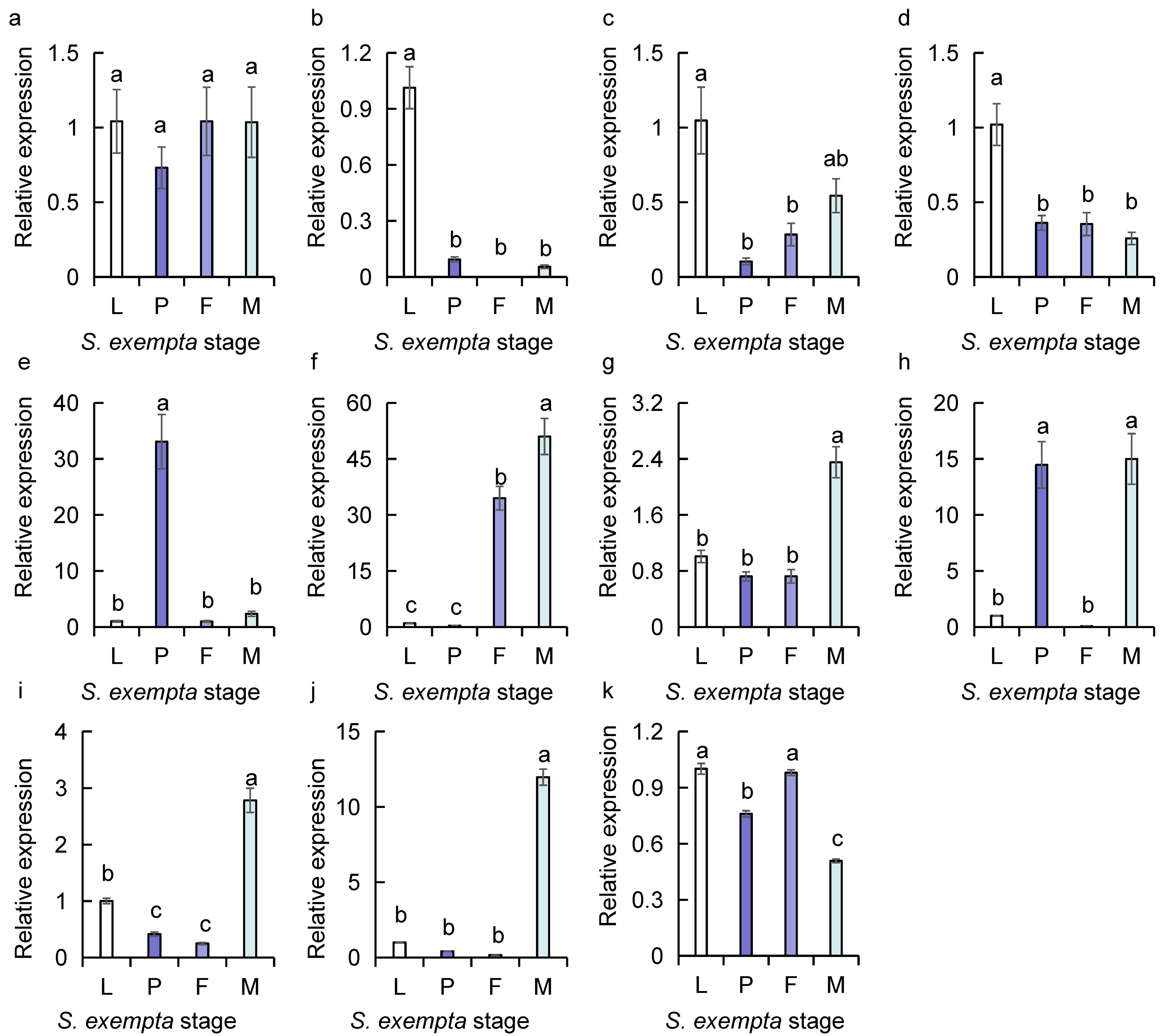

Supplement: Supplemental Information 2 — (a) SexeOBP 9 (F = 0.555, d.f. =3,8, P = 0.659); (b) SexeOBP 13 (F = 72.571, d.f. =3,8, P = 0.000); (c) SexeOBP 15 (F = 9.771, d.f. =3,8, P = 0.005); (d) SexeOBP 16 (F = 16.651, d.f. =3,8, P = 0.001); (e) SexeOBP 18 (F = 42.290, d.f. =3,8, P = 0.000); (f) SexeOBP 26 (F = 76.012, d.f. =3,8, P = 0.000); (g) SexeOBP 29 (F = 34.62, d.f. =3,8, P = 0.000); (h) SexeOBP 30 (F = 28.574, d.f. =3,8, P = 0.000); (i) SexeOBP 31 (F = 107.519, d.f. =3,8, P = 0.000); (j) SexeOBP 35 (F = 459.605, d.f. =3,8, P = 0.000); (k) SexeOBP 37 (F = 152.93, d.f. =3,8, P = 0.000). Mean ± SE. Different letters showed significant difference (one-way ANOVA). [file peerj-09-12132-s002.png]

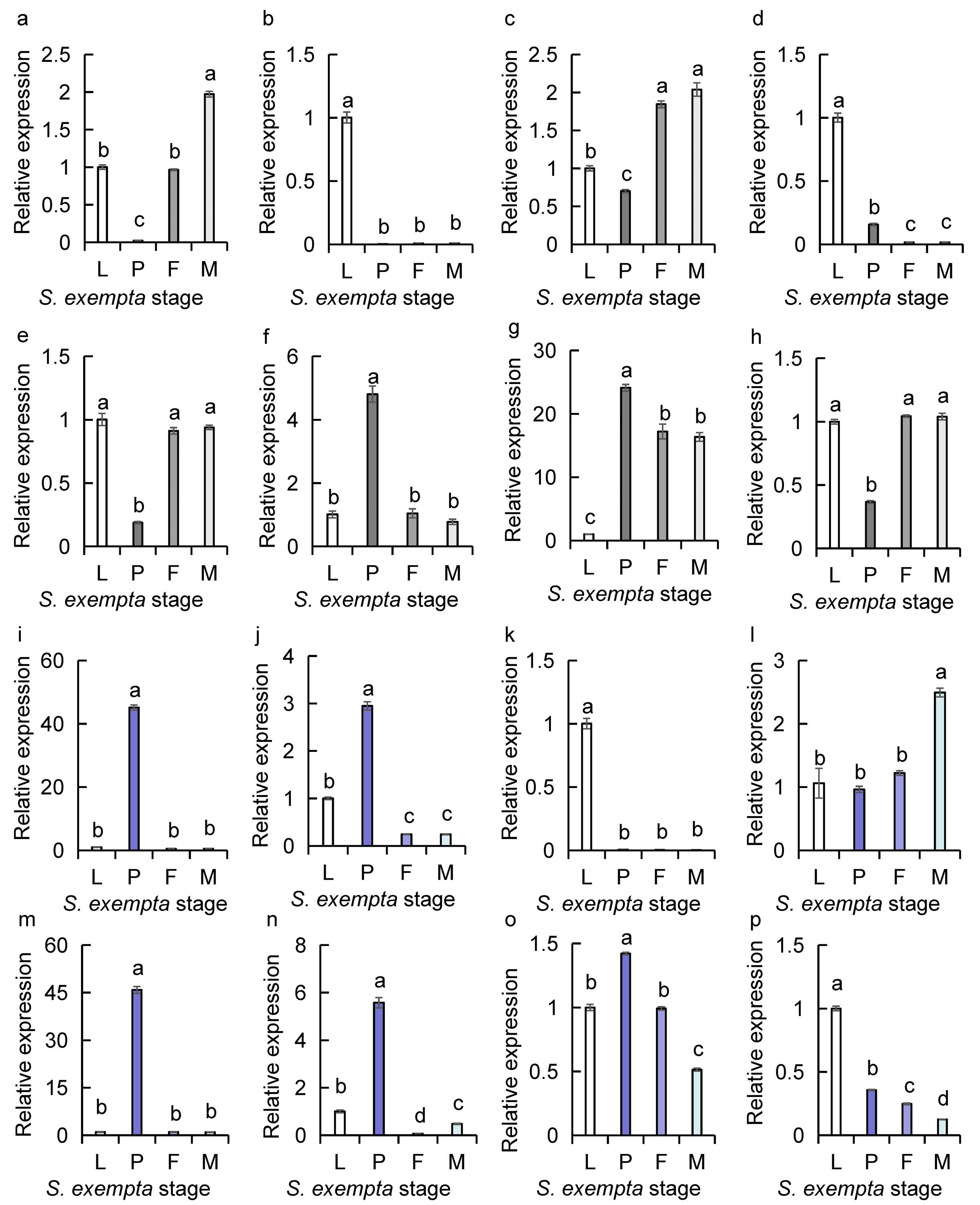

Supplement: Supplemental Information 3 — (a) SexeCSP 2 (F = 1037.806, d.f. =3,8, P = 0.000); (b) SexeCSP 4 (F = 523.254, d.f. =3,8, P = 0.000); (c) SexeCSP 5 (F = 147.502, d.f. =3,8, P = 0.000); (d) SexeOBP 8 (F = 663.712, d.f. =3,8, P = 0.000); (e) SexeCSP 9 (F = 179.16, d.f. =3,8, P = 0.000); (f) SexeCSP 10 (F = 145.377, d.f. =3,8, P = 0.000); (g) SexeCSP 12 (F = 180.618, d.f. =3,8, P = 0.000); (h) SexeCSP 13 (F = 359.123, d.f. =3,8, P = 0.000); (i) SexeCSP 17 (F = 3131.788, d.f. =3,8, P = 0.000); (j) SexeCSP 19 (F = 759.292, d.f. =3,8, P = 0.000); (k) SexeCSP 23 (F = 587.196, d.f. =3,8, P = 0.000); (l) SexeCSP 25 (F = 31.917, d.f. =3,8, P = 0.000); (m) SexeCSP 26 (F = 1734.3, d.f. =3,8, P = 0.000); (n) SexeCSP 28 (F = 537.055, d.f. =3,8, P = 0.000); (o) SexeCSP 30 (F = 580.952, d.f. =3,8, P = 0.000); (p) SexeCSP 33 (F = 1583.71, d.f. =3,8, P = 0.000). Mean ± SE. Different letters showed significant difference (one-way ANOVA). [file peerj-09-12132-s003.png]
