# Supplementary material for: Sex- and stage-dependent expression patterns of odorant-binding and chemosensory protein genes in Spodoptera exempta
Source: PeerJ. 2021 Sep 13;9:e12132. doi: 10.7717/peerj.12132 (PMC8445084; doi:10.7717/peerj.12132)
Supplement: Supplemental Information 4 [file peerj-09-12132-s004.doc]

Table S1 Data output for each pool of *S. exempta*.

| **Sample_ID** | **Stage** | **Total_Reads** | **Total_Bases** | **Library accession** |
| --- | --- | --- | --- | --- |
| SEAF19 | Female adults | 52195732 | 7837811416 | SRR8594176 |
| SEAF20 |  | 56120828 | 8427549517 |  |
| SEAF21 |  | 58071458 | 8718708253 |  |
| V1V2SEAF22 |  | 58183990 | 8738435672 | SRR8594175 |
| V1V2SEAF23 |  | 53619788 | 8050899805 |  |
| V1V2SEAF24 |  | 55936350 | 8394699051 |  |
| V1V2SEAM16 | Male adults | 59726944 | 8961911923 | SRR8594173 |
| V1V2SEAM17 |  | 51132448 | 7665355542 |  |
| V1V2SEAM18 |  | 54891614 | 8233667718 |  |
| SEAM13 |  | 54128618 | 8121616770 | SRR8594174 |
| SEAM14 |  | 55062672 | 8255777449 |  |
| SEAM15 |  | 59801452 | 8967832093 |  |
| SEL1 | Larvae | 61444862 | 9218295348 | SRR8594178 |
| SEL2 |  | 49513190 | 7424395191 |  |
| SEL3 |  | 55586150 | 8338493570 |  |
| V1V2SEL4 |  | 58120948 | 8722500233 | SRR8594177 |
| V1V2SEL5 |  | 61555154 | 9230262186 |  |
| V1V2SEL6 |  | 50698282 | 7605955856 |  |
| V1V2SEP10 | Pupae | 53404358 | 8011065327 | SRR8594179 |
| V1V2SEP11 |  | 52452342 | 7865742529 |  |
| V1V2SEP12 |  | 52112708 | 7814678815 |  |
| 8-Sep |  | 49173004 | 7369872703 | SRR8594180 |
| 9-Sep |  | 56255404 | 8428309273 |  |
