# Supplementary material for: Sex- and stage-dependent expression patterns of odorant-binding and chemosensory protein genes in Spodoptera exempta
Source: PeerJ. 2021 Sep 13;9:e12132. doi: 10.7717/peerj.12132 (PMC8445084; doi:10.7717/peerj.12132)
Supplement: Supplemental Information 6 [file peerj-09-12132-s006.docx]

Table S3 The OBPs identified in *S. exempta* by transcriptome

| Unigene | Gene name | ORF(aa) | SP | Mw(kDa) | pI | Identity | E-value | Annotation |
| --- | --- | --- | --- | --- | --- | --- | --- | --- |
| TRINITY_DN43257_c0_g1 | SexeOBP1 | 136 | 19 | 15.4 | 7.63 | 89 | 1.4E-65 | XP_022826781.1 GOBP lush-like [Spodoptera litura] |
| TRINITY_DN47412_c0_g1 | SexeOBP2 | 145 | 24 | 15.63 | 8.28 | 93.5 | 1.7E-69 | AKI87969.1 OBP 8 [Spodoptera litura] |
| TRINITY_DN51667_c0_g1 | SexeOBP3 | 138 | 18 | 15.11 | 3.95 | 34.5 | 0.0000069 | XP_021196071.1 GOBP 56d-like [Helicoverpa armigera] |
| TRINITY_DN50125_c0_g1 | SexeOBP4 | 145 | 24 | 16.28 | 7.95 | 92.2 | 3.3E-70 | ALD65891.1 OBP 17 [Spodoptera litura] |
| TRINITY_DN47877_c0_g1 | SexeOBP5 | 154 | 23 | 16.9 | 4.95 | 76.6 | 4.3E-64 | XP_021194665.1 GOBP 19d-like [Helicoverpa armigera] |
| TRINITY_DN18520_c0_g1 | SexeOBP6 | 147 | 19 | 16.54 | 4.65 | 65.1 | 1.3E-50 | ALJ30198.1 putative OBP11 [Spodoptera litura] |
| TRINITY_DN50402_c0_g1 | SexeOBP7 | 148 | 19 | 16.8 | 5.68 | 87.8 | 4.3E-71 | XP_022826775.1 GOBP 28a-like X4 [Spodoptera litura] |
| TRINITY_DN53358_c0_g1 | SexeOBP8 | 147 | 20 | 16.45 | 4.55 | 84.4 | 9.2E-67 | ALJ30188.1 putative OBP1 [Spodoptera litura] |
| TRINITY_DN50465_c0_g1 | SexeOBP9 | 157 | 20 | 17.54 | 4.31 | 94.3 | 2.6E-78 | ADY17882.1 OBP [Spodoptera exigua] |
| TRINITY_DN116382_c0_g1 | SexeOBP10 | 92 | 20 | 6.81 | 10.12 | 51.7 | 0.0000001 | ARO70177.1 OBP18 [Dendrolimus punctatus] |
| TRINITY_DN50958_c0_g1 | SexeOBP11 | 142 | 21 | 16.28 | 8.82 | 96.5 | 1.2E-71 | AGH70103.1 OBP 7 [Spodoptera exigua] |
| TRINITY_DN58222_c2_g2 | SexeOBP12 | 146 | 19 | 13.03 | 5.92 | 50 | 4.2E-23 | ARO70177.1 OBP 18 [Dendrolimus punctatus] |
| TRINITY_DN52285_c0_g1 | SexeOBP13 | 149 | 22 | 16.34 | 4.73 | 87.2 | 1.2E-68 | AKT26503.1 OBP 26 [Spodoptera exigua] |
| TRINITY_DN46842_c0_g1 | SexeOBP14 | 148 | 18 | 16.41 | 5.26 | 64.2 | 2E-49 | ADY17885.1 OBP [Spodoptera exigua] |
| TRINITY_DN59759_c0_g1 | SexeOBP15 | 81 | 21 | 8.45 | 6.22 | 89.9 | 1.2E-30 | AAR28762.1 OBP [Spodoptera frugiperda] |
| TRINITY_DN50679_c0_g1 | SexeOBP16 | 157 | 32 | 16.09 | 4.3 | 37.2 | 7.9E-22 | AGR39565.1 OBP 2 [Agrotis ipsilon] |
| TRINITY_DN49331_c0_g1 | SexeOBP17 | 158 | 25 | 17.92 | 8.49 | 92.4 | 1.3E-80 | XP_022826771.1 GOBP 28a-like [Spodoptera litura] |
| TRINITY_DN56406_c1_g1 | SexeOBP18 | 133 | 16 | 15.07 | 9.21 | 95.5 | 2.1E-66 | AGH70105.1 OBP 9 [Spodoptera exigua] |
| TRINITY_DN148967_c0_g1 | SexeOBP19 | 124 | 17 | 13.39 | 8.37 | 84.4 | 1.1E-52 | ADY17884.1 OBP [Spodoptera exigua] |
| TRINITY_DN41139_c0_g1 | SexeOBP20 | 149 | 26 | 17.24 | 4.39 | 98 | 5.6E-80 | AGH70104.1 OBP 8 [Spodoptera exigua] |
| TRINITY_DN54627_c0_g2 | SexeOBP21 | 153 | 21 | 17.03 | 4.37 | 73.9 | 1.1E-59 | ASA40072.1 OBP 33 [Helicoverpa assulta] |
| TRINITY_DN51500_c0_g1 | SexeOBP22 | 145 | 24 | 16.89 | 8.04 | 93.8 | 3E-74 | XP_022826780.1 GOBP 72-like [Spodoptera litura] |
| TRINITY_DN26993_c0_g1 | SexeOBP23 | 135 | 18 | 12.87 | 7.81 | 73.2 | 1.3E-42 | KOB73305.1 OBP [Operophtera brumata] |
| TRINITY_DN46265_c0_g2 | SexeOBP24 | 153 | 21 | 16.36 | 4.25 | 58.9 | 7.1E-40 | ALJ30193.1 putative OBP6 [Spodoptera litura] |
| TRINITY_DN150426_c0_g1 | SexeOBP25 | 86 | 21 | 9.07 | 5.08 | 98.8 | 5.3E-39 | AKI87967.1 OBP 6 [Spodoptera litura] |
| TRINITY_DN53432_c1_g2 | SexeOBP26 | 117 | 21 | 13.01 | 4.22 | 81 | 7.3E-46 | AKI87966.1 OBP 5 [Spodoptera litura] |
| TRINITY_DN47444_c0_g1 | SexeOBP27 | 145 | 17 | 16.27 | 7.54 | 90.3 | 3.2E-38 | ADY17886.1 OBP [Spodoptera exigua] |
| TRINITY_DN55163_c0_g1 | SexeOBP28 | 336 | 20 | 38.95 | 7.05 | 94.3 | 5.8E-112 | ALD65883.1 OBP 9 [Spodoptera litura] |
| TRINITY_DN61103_c2_g1 | SexeOBP29 | 157 | 19 | 18.29 | 7.78 | 82.2 | 2E-69 | KOB73304.1 OBP [Operophtera brumata] |
| TRINITY_DN53775_c0_g1 | SexeOBP30 | 198 | NA | 15.91 | 8.28 | 92.5 | 6.3E-70 | AKT26499.1 OBP 22 [Spodoptera exigua] |
| TRINITY_DN56221_c1_g1 | SexeOBP31 | 212 | NA | 19.75 | 5.02 | 78.9 | 7.2E-73 | NP_001140186.1 OBP 2 precursor [Bombyx mori] |
| TRINITY_DN49634_c0_g1 | SexeOBP32 | 177 | NA | 20.78 | 9.29 | 74 | 2E-67 | ARO70197.1 OBP 38 [Dendrolimus punctatus] |
| TRINITY_DN28131_c0_g1 | SexeOBP33 | 143 | NA | 15.14 | 8.14 | 63.8 | 3.2E-45 | NP_001140187.1 OBP 3 precursor [Bombyx mori] |
| TRINITY_DN70250_c0_g1 | SexeOBP34 | 132 | NA | 12.08 | 7.93 | 81.7 | 8.8E-46 | ADY17884.1 OBP [Spodoptera exigua] |
| TRINITY_DN56323_c9_g2 | SexeOBP35 | 114 | NA | 16.41 | 9.05 | 79 | 9.4E-58 | AIL54057.1 OBP 21, partial [Chilo suppressalis] |
| TRINITY_DN52069_c0_g1 | SexeOBP36 | 182 | NA | 20.41 | 8.7 | 83 | 3.2E-81 | ARO70198.1 OBP 39 [Dendrolimus punctatus] |
| TRINITY_DN52143_c0_g4 | SexeOBP37 | 119 | NA | 13.05 | 8.31 | 70.7 | 3.2E-41 | ADY17884.1 OBP [Spodoptera exigua] |
| TRINITY_DN49162_c0_g1 | SexeOBP38 | 124 | NA | 13.93 | 4.14 | 37 | 1.7E-18 | AGC92789.1 OBP 9 [Helicoverpa assulta] |
| TRINITY_DN142191_c0_g1 | SexeOBP39 | 70 | NA | 7.89 | 4.22 | 100 | 5.9E-32 | AKI87966.1 OBP 5 [Spodoptera litura] |
| TRINITY_DN59759_c0_g7 | SexeOBP40 | 77 | NA | 8.47 | 4.87 | 100 | 8.9E-35 | AAR28762.1 OBP [Spodoptera frugiperda] |
