# Supplementary material for: Sex- and stage-dependent expression patterns of odorant-binding and chemosensory protein genes in Spodoptera exempta
Source: PeerJ. 2021 Sep 13;9:e12132. doi: 10.7717/peerj.12132 (PMC8445084; doi:10.7717/peerj.12132)
Supplement: Supplemental Information 7 [file peerj-09-12132-s007.docx]

Table S4 The CSPs identified in *S. exempta* by transcriptome

| Unigene | Gene name | ORF(aa) | SP | Mw(kDa) | pI | Identity | E-value | Annotation |
| --- | --- | --- | --- | --- | --- | --- | --- | --- |
| TRINITY_DN40377_c0_g1 | SexeCSP1 | 126 | 15 | 13.96 | 7.94 | 80.8 | 3.23E-42 | AKT26488.1 chemosensory protein 12 [Spodoptera exigua] |
| TRINITY_DN58582_c3_g1 | SexeCSP2 | 69 | 18 | 7.45 | 7.25 | 95.4 | 1.37E-21 | ALJ30214.1 putative chemosensory protein CSP3 [Spodoptera litura] |
| TRINITY_DN84988_c0_g1 | SexeCSP3 | 126 | 15 | 14.18 | 6.92 | 88 | 7.02E-41 | AKT26488.1 chemosensory protein 12 [Spodoptera exigua] |
| TRINITY_DN58849_c0_g1 | SexeCSP4 | 123 | 16 | 13.83 | 8.6 | 87 | 5.49E-44 | AKT26489.1 chemosensory protein 13 [Spodoptera exigua] |
| TRINITY_DN58168_c1_g2 | SexeCSP5 | 148 | 21 | 16.83 | 4.77 | 76.9 | 1.77E-49 | AND82446.1 chemosensory protein 4 [Athetis dissimilis] |
| TRINITY_DN56114_c4_g5 | SexeCSP6 | 107 | 22 | 12 | 9.53 | 91.6 | 2.41E-11 | AKT26485.1 chemosensory protein 8 [Spodoptera exigua] |
| TRINITY_DN36094_c0_g1 | SexeCSP7 | 122 | 16 | 14.22 | 6.34 | 89.7 | 3.27E-48 | AAY26143.1 chemosensory protein CSP [Spodoptera litura] |
| TRINITY_DN50809_c0_g1 | SexeCSP8 | 122 | 18 | 13.68 | 4.92 | 89.3 | 3.38E-40 | AKT26491.1 chemosensory protein 16 [Spodoptera exigua] |
| TRINITY_DN61504_c9_g1 | SexeCSP9 | 65 | NA | 7.69 | 5.56 | 83.6 | 2.03E-19 | ALJ30219.1 putative chemosensory protein CSP8 [Spodoptera litura] |
| TRINITY_DN51508_c0_g1 | SexeCSP10 | 56 | 18 | 6.37 | 7.25 | 96.4 | 1.91E-15 | ABM67689.1 chemosensory protein CSP2 [Spodoptera exigua] |
| TRINITY_DN86597_c0_g1 | SexeCSP11 | 86 | 16 | 9.54 | 7.81 | 100 | 8.41E-31 | ALJ30223.1 putative chemosensory protein CSP12 [Spodoptera litura] |
| TRINITY_DN58311_c2_g2 | SexeCSP12 | 288 | 16 | 32.01 | 10.11 | 92.7 | 1.5E-42 | AKT26490.1 chemosensory protein 14 [Spodoptera exigua] |
| TRINITY_DN61504_c9_g2 | SexeCSP13 | 81 | NA | 10.69 | 6.25 | 96.8 | 2.54E-35 | ALJ30219.1 putative chemosensory protein CSP8 [Spodoptera litura] |
| TRINITY_DN2896_c0_g1 | SexeCSP14 | 113 | 16 | 12.69 | 6.5 | 86.7 | 1.51E-09 | ALJ30218.1 putative chemosensory protein CSP7 [Spodoptera litura] |
| TRINITY_DN57756_c0_g3 | SexeCSP15 | 123 | 18 | 13.58 | 5.16 | 82.9 | 5.82E-31 | AKT26481.1 chemosensory protein 4 [Spodoptera exigua] |
| TRINITY_DN15026_c0_g1 | SexeCSP16 | 84 | 20 | 9.14 | 8.62 | 100 | 5.54E-08 | ALJ30220.1 putative chemosensory protein CSP9 [Spodoptera litura] |
| TRINITY_DN56075_c0_g2 | SexeCSP17 | 69 | 16 | 7.33 | 9.82 | 90.6 | 3.86E-19 | AKT26487.1 chemosensory protein 11 [Spodoptera exigua] |
| TRINITY_DN14892_c0_g1 | SexeCSP18 | 110 | 17 | 12.27 | 6.5 | 90 | 3.36E-29 | AKT26493.1 chemosensory protein 19 [Spodoptera exigua] |
| TRINITY_DN58168_c2_g1 | SexeCSP19 | 128 | 18 | 14.73 | 9.19 | 88.2 | 1.55E-46 | ABM67688.1 chemosensory protein CSP1 [Spodoptera exigua] |
| TRINITY_DN61237_c0_g1 | SexeCSP20 | 122 | 21 | 13.95 | 9.52 | 99.2 | 5.09E-35 | AKT26486.1 chemosensory protein 10 [Spodoptera exigua] |
| TRINITY_DN46377_c0_g1 | SexeCSP21 | 123 | 16 | 13.73 | 8.84 | 97.6 | 1.8E-41 | ALJ30217.1 putative chemosensory protein CSP6 [Spodoptera litura] |
| TRINITY_DN46377_c0_g2 | SexeCSP22 | 123 | 16 | 13.81 | 8.6 | 94.3 | 1.14E-41 | ALJ30217.1 putative chemosensory protein CSP6 [Spodoptera litura] |
| TRINITY_DN57687_c0_g1 | SexeCSP23 | 122 | 17 | 13.83 | 5.76 | 86.1 | 8.32E-31 | AKT26493.1 chemosensory protein 19 [Spodoptera exigua] |
| TRINITY_DN55721_c0_g1 | SexeCSP24 | 153 | NA | 17.15 | 8.62 | 60.8 | 3.48E-11 | BAV56818.1 chemosensory protein 14 [Ostrinia furnacalis] |
| TRINITY_DN60763_c0_g1 | SexeCSP25 | 127 | 17 | 14.6 | 8.2 | 93.7 | 2.39E-52 | AKT26483.1 chemosensory protein 6 [Spodoptera exigua] |
| TRINITY_DN63121_c1_g1 | SexeCSP26 | 124 | 16 | 14.35 | 7.76 | 72.6 | 9.47E-46 | AAY26143.1 chemosensory protein CSP [Spodoptera litura] |
| TRINITY_DN139736_c0_g1 | SexeCSP27 | 114 | 16 | 12.92 | 4.78 | 66.7 | 5.06E-07 | ALJ30218.1 putative chemosensory protein CSP7 [Spodoptera litura] |
| TRINITY_DN57756_c0_g1 | SexeCSP28 | 123 | 18 | 13.65 | 5.5 | 80.5 | 4.68E-31 | AND82448.1 chemosensory protein 6 [Athetis dissimilis] |
| TRINITY_DN57756_c0_g2 | SexeCSP29 | 75 | NA | 12.48 | 4.88 | 81.6 | 8.87E-31 | AKT26481.1 chemosensory protein 4 [Spodoptera exigua] |
| TRINITY_DN60392_c1_g3 | SexeCSP30 | 63 | NA | 7.6 | 8.19 | 96.8 | 3.81E-18 | ABM67689.1 chemosensory protein CSP2 [Spodoptera exigua] |
| TRINITY_DN56075_c0_g1 | SexeCSP31 | 105 | 13 | 11.82 | 8.48 | 98.1 | 9.45E-45 | AKT26487.1 chemosensory protein 11 [Spodoptera exigua] |
| TRINITY_DN129070_c0_g1 | SexeCSP32 | 73 | NA | 8.58 | 9.6 | 91.8 | 1.24E-24 | ABM67688.1 chemosensory protein CSP1 [Spodoptera exigua] |
| TRINITY_DN61349_c1_g1 | SexeCSP33 | 65 | AN | 7.51 | 8.49 | 72.3 | 5.34E-17 | AKT26488.1 chemosensory protein 12 [Spodoptera exigua] |
