# Supplementary material for: Sex- and stage-dependent expression patterns of odorant-binding and chemosensory protein genes in Spodoptera exempta
Source: PeerJ. 2021 Sep 13;9:e12132. doi: 10.7717/peerj.12132 (PMC8445084; doi:10.7717/peerj.12132)
Supplement: Supplemental Information 8 [file peerj-09-12132-s008.docx]

Table S5 Conserved C-Pattern in OBPs of *S. exempta*

| Gene Name | C-Pattern |
| --- | --- |
| SexeOBP1 | C1-X_26_-C2-X_3_-C3-X_40_-C4-X_10_-C5-X_8_-C6 |
| SexeOBP2 | C1-X_26_-C2-X_3_-C3-X_40_-C4-X_10_-C5-X_8_-C6 |
| SexeOBP4 | C1-X_26_-C2-X_3_-C3-X_40_-C4-X_10_-C5-X_8_-C6 |
| SexeOBP5 | C1-X_27_-C2-X_3_-C3-X_42_-C4-X_14_-C5-X_8_-C6 |
| SexeOBP6 | C1-X_24_-C2-X_3_-C3-X_45_-C4-X_15_-C5-X_8_-C6 |
| SexeOBP7 | C1-X_26_-C2-X_3_-C3-X_43_-C4-X_14_-C5-X_8_-C6 |
| SexeOBP8 | C1-X_26_-C2-X_3_-C3-X_43_-C4-X_14_-C5-X_8_-C6 |
| SexeOBP9 | C1-X_29_-C2-X_3_-C3-X_42_-C4-X_9_-C5-X_8_-C6 |
| SexeOBP11 | C1-X_26_-C2-X_3_-C3-X_40_-C4-X_10_-C5-X_8_-C6 |
| SexeOBP13 | C1-X_25_-C2-X_3_-C3-X_40_-C4-X_14_-C5-X_8_-C6 |
| SexeOBP14 | C1-X_26_-C2-X_3_-C3-X_40_-C4-X_14_-C5-X_8_-C6 |
| SexeOBP16 | C1-X_25_-C2-X_3_-C3-X_39_-C4-X_14_-C5-X_8_-C6 |
| SexeOBP17 | C1-X_26_-C2-X_3_-C3-X_41_-C4-X_14_-C5-X_8_-C6 |
| SexeOBP20 | C1-X_26_-C2-X_3_-C3-X_37_-C4-X_10_-C5-X_8_-C6 |
| SexeOBP21 | C1-X_26_-C2-X_3_-C3-X_42_-C4-X_14_-C5-X_8_-C6 |
| SexeOBP22 | C1-X_26_-C2-X_3_-C3-X_41_-C4-X_10_-C5-X_8_-C6 |
| SexeOBP23 | C1-X_19_-C2-X_3_-C3-X_41_-C4-X_13_-C5-X_8_-C6 |
| SexeOBP24 | C1-X_25_-C2-X_3_-C3-X_41_-C4-X_13_-C5-X_8_-C6 |
| SexeOBP27 | C1-X_26_-C2-X_3_-C3-X_43_-C4-X_13_-C5-X_8_-C6 |
| SexeOBP31 | C1-X_26_-C2-X_3_-C3-X_42_-C4-X_10_-C5-X_8_-C6 |
| SexeOBP32 | C1-X_26_-C2-X_3_-C3-X_42_-C4-X_10_-C5-X_8_-C6 |
| SexeOBP33 | C1-X_25_-C2-X_3_-C3-X_42_-C4-X_11_-C5-X_8_-C6 |
| SexeOBP35 | C1-X_26_-C2-X_3_-C3-X_42_-C4-X_10_-C5-X_8_-C6 |
| SexeOBP36 | C1-X_26_-C2-X_3_-C3-X_42_-C4-X_10_-C5-X_8_-C6 |
| SexeOBP3 | C1-X_28_-C3-X_39_-C4-X_17_- C6 |
| SexeOBP12 | C1-X_34_-C3-X_36_-C4-X_17_- C6 |
| SexeOBP18 | C1-X_31_-C3-X_36_-C4-X_17_- C6 |
| SexeOBP19 | C1-X_30_-C3-X_36_-C4-X_17_- C6 |
| SexeOBP30 | C1-X_30_-C3-X_38_-C4-X_17_- C6 |
| SexeOBP34 | C1-X_30_-C3-X_39_-C4-X_17_- C6 |
