# Supplementary material for: Sex- and stage-dependent expression patterns of odorant-binding and chemosensory protein genes in Spodoptera exempta
Source: PeerJ. 2021 Sep 13;9:e12132. doi: 10.7717/peerj.12132 (PMC8445084; doi:10.7717/peerj.12132)
Supplement: Supplemental Information 9 [file peerj-09-12132-s009.docx]

Table S6 GenBank accession numbers of insect CSP genes used in the phylogenetic analysis

| **Species** | **Gene name** | **Acc. no.** |
| --- | --- | --- |
| *Bombyx mori* | BmorCSP1 | ABH88194 |
|  | BmorCSP2 | ABH88195 |
|  | BmorCSP3 | ABH88196 |
|  | BmorCSP4 | ABH88197 |
|  | BmorCSP5 | ABH88198 |
|  | BmorCSP6 | ABH88199 |
|  | BmorCSP7 | ABH88200 |
|  | BmorCSP8 | ABH88201 |
|  | BmorCSP9 | ABH88202 |
|  | BmorCSP10 | ABH88203 |
|  | BmorCSP11 | ABH88204 |
|  | BmorCSP12 | ABH88205 |
|  | BmorCSP13 | ABH88206 |
|  | BmorCSP14 | ABH88207 |
|  | BmorCSP15 | ABH88208 |
|  | BmorCSP16 | ABH88209 |
| *Plutella xylostella* | PxylCSP1 | Yang et al. (2017) Scientific Reports 7: 11941 |
|  | PxylCSP2 |  |
|  | PxylCSP3 |  |
|  | PxylCSP4 |  |
|  | PxylCSP5 |  |
|  | PxylCSP6 |  |
|  | PxylCSP7 |  |
|  | PxylCSP8 |  |
|  | PxylCSP9 |  |
|  | PxylCSP10 |  |
|  | PxylCSP11 |  |
|  | PxylCSP12 |  |
|  | PxylCSP13 |  |
|  | PxylCSP14 |  |
|  | PxylCSP15 |  |
| *Helicoverpa armigera* | HarmCSP | AAK53762 |
|  | HarmCSP2 | AEX07265 |
|  | HarmCSP3 | AEX07266 |
|  | HarmCSP4 | AEX07269 |
|  | HarmCSP5 | AEB54579 |
|  | HarmCSP6 | AEX07267 |
|  | HarmCSP7 | AEX07268 |
| *Papilio xuthus* | PxutCSP1 | AB260116 |
|  | PxutCSP2 | AB260117 |
|  | PxutCSP3 | AB260118 |
|  | PxutCSP4 | AB430771 |
|  | PxutCSP5 | AB260120 |
|  | PxutCSP6 | AB260121 |
|  | PxutCSP7 | AB260122 |
|  | PxutCSP8 | AB260124 |
|  | PxutCSP9 | AB260125 |
|  | PxutCSP10 | AB260126 |
|  | PxutCSP11 | AB430775 |
|  | PxutCSP12 | AB430777 |
|  | PxutCSP13 | AB430778 |
| *Cnaphalocrocis medinalis* | CmedCSP1 | AGI37361 |
|  | CmedCSP2 | AGI37363 |
|  | CmedCSP3 | AGI37365 |
|  | CmedCSP4 | KM365188 |
|  | CmedCSP5 | KM365189 |
|  | CmedCSP6 | KM365190 |
|  | CmedCSP7 | KM365191 |
|  | CmedCSP8 | KM365192 |
|  | CmedCSP9 | KM365193 |
|  | CmedCSP10 | KM365194 |
|  | CmedCSP11 | KM365195 |
|  | CmedCSP12 | KM365196 |
|  | CmedCSP13 | KM365197 |
|  | CmedCSP14 | KM365198 |
|  | CmedCSP15 | KM365199 |
|  | CmedCSP16 | KM365200 |
|  | CmedCSP17 | KM365201 |
|  | CmedCSP18 | KM365202 |
|  | CmedCSP19 | KM365203 |
|  | CmedCSP20 | KM365204 |
|  | CmedCSP21 | KM365205 |
|  | CmedCSP22 | KM365206 |
