# Supplementary material for: Sex- and stage-dependent expression patterns of odorant-binding and chemosensory protein genes in Spodoptera exempta
Source: PeerJ. 2021 Sep 13;9:e12132. doi: 10.7717/peerj.12132 (PMC8445084; doi:10.7717/peerj.12132)
Supplement: Supplemental Information 10 — L = larvae, P = pupae, M = male adults, F = female adults. [file peerj-09-12132-s010.docx]

Table S7 The TPM values of *S. exempta* OBPs in different samples. L = larvae, P = pupae, M = male adults, F = female adults.

| Gene name | L1 | L2 | L3 | L4 | L5 | L6 | P1 | P2 | P3 | P4 | P5 | M1 | M2 | M3 | M4 | M5 | M6 | F1 | F2 | F3 | F4 | F5 | F6 |
| --- | --- | --- | --- | --- | --- | --- | --- | --- | --- | --- | --- | --- | --- | --- | --- | --- | --- | --- | --- | --- | --- | --- | --- |
| SexeOBP1 | 1.22 | 1.64 | 2.25 | 0.54 | 1.31 | 0.97 | 0 | 0 | 0 | 0 | 0 | 0 | 0 | 0 | 0 | 0 | 0 | 0.26 | 0 | 0 | 0 | 0 | 0 |
| SexeOBP2 | 0 | 0 | 0 | 0 | 0 | 0 | 0 | 0 | 0 | 0 | 0 | 1.64 | 1.86 | 0.75 | 1.76 | 1.26 | 0.52 | 0.75 | 1.59 | 0.17 | 0.72 | 0 | 0.63 |
| SexeOBP3 | 0 | 0 | 0 | 0 | 0 | 0 | 0 | 0.13 | 0 | 0 | 0 | 3.23 | 3.94 | 6.2 | 6.87 | 8.22 | 5.35 | 0 | 0 | 0 | 0 | 0.08 | 0 |
| SexeOBP4 | 0 | 0 | 0 | 0.16 | 0 | 0 | 0 | 0 | 0 | 0 | 0 | 5.21 | 2.4 | 3.65 | 2.63 | 3.99 | 1.11 | 0.21 | 0.39 | 0 | 2.53 | 1.9 | 0.82 |
| SexeOBP5 | 0.58 | 0.92 | 1.58 | 0.93 | 1.04 | 1.44 | 0 | 0 | 0.24 | 0 | 0 | 0 | 0.12 | 0.11 | 0 | 0 | 0 | 0 | 0.38 | 0 | 0 | 0.14 | 0 |
| SexeOBP6 | 1.04 | 0 | 0.16 | 0.31 | 0.56 | 0.5 | 0 | 0 | 0 | 0 | 0 | 0 | 0 | 0 | 0.16 | 0 | 0 | 0 | 0 | 0 | 0 | 0 | 0 |
| SexeOBP7 | 1.05 | 0.14 | 0.25 | 0.25 | 0.34 | 1.31 | 0.14 | 0 | 0 | 0 | 0 | 0 | 0 | 0 | 0.25 | 0 | 0 | 0 | 0.15 | 0 | 0 | 0.16 | 0 |
| SexeOBP8 | 0.38 | 0.11 | 0.42 | 0.2 | 0.37 | 0 | 0.11 | 0.43 | 0 | 0.24 | 1.07 | 7.72 | 4.9 | 5.91 | 5.13 | 5.18 | 5.7 | 1.88 | 1.01 | 0.61 | 3.22 | 1.74 | 1.56 |
| SexeOBP9 | 0.38 | 0.28 | 0.34 | 0.33 | 0.23 | 0.27 | 0 | 0 | 0 | 0 | 0 | 9.4 | 12.47 | 6.3 | 10.23 | 9.61 | 4.52 | 2.39 | 1.72 | 0.1 | 4.4 | 3.35 | 2.61 |
| SexeOBP10 | 0 | 0 | 0 | 0 | 0 | 0 | 0 | 0 | 0 | 0 | 0 | 0 | 0 | 0 | 0 | 0 | 0 | 0 | 0 | 0 | 0 | 0 | 0 |
| SexeOBP11 | 0.8 | 1.41 | 1.35 | 0.53 | 1.17 | 0.5 | 0 | 0 | 0 | 0 | 0 | 2.41 | 3.84 | 1.71 | 6.33 | 4.44 | 4.57 | 1.58 | 0.9 | 0.08 | 1.77 | 2.69 | 0.92 |
| SexeOBP12 | 0 | 0 | 0 | 0 | 0 | 0 | 0.1 | 0.1 | 0.04 | 0 | 0 | 5.92 | 3.69 | 6.93 | 5.27 | 4.75 | 4.1 | 0 | 0.03 | 0 | 0 | 0 | 0 |
| SexeOBP13 | 4.56 | 14.15 | 9.67 | 25.13 | 14.76 | 6.99 | 0.16 | 0 | 0 | 0.25 | 0.25 | 0 | 0 | 0 | 0 | 0 | 0 | 0 | 0 | 0 | 0 | 0 | 0 |
| SexeOBP14 | 0.88 | 0.3 | 1.72 | 4.72 | 1.9 | 1.84 | 0 | 0 | 0 | 0 | 0 | 0 | 0 | 0 | 0 | 0 | 0 | 0 | 0 | 0 | 0 | 0 | 0 |
| SexeOBP15 | 113.67 | 161.95 | 188.13 | 287.86 | 155.47 | 132.79 | 0.19 | 0.13 | 0.04 | 0.02 | 0.18 | 36.48 | 29.61 | 26.07 | 35.13 | 30.43 | 27.75 | 14.94 | 15.72 | 14.76 | 24.04 | 21.15 | 18.13 |
| SexeOBP16 | 4.08 | 12.35 | 12.34 | 17.22 | 6.98 | 8.24 | 0.69 | 0 | 0 | 0.18 | 0.09 | 0.15 | 0.15 | 0.54 | 0.24 | 0.15 | 0.18 | 0.34 | 0 | 0 | 0.25 | 0.2 | 0.33 |
| SexeOBP17 | 0.24 | 0 | 0.91 | 1.64 | 0.23 | 0 | 0 | 0 | 0 | 0 | 0 | 0 | 0.72 | 0.13 | 0.26 | 0.3 | 0.73 | 0 | 0.31 | 0 | 0 | 0 | 0.16 |
| SexeOBP18 | 3.28 | 4.69 | 5.3 | 8.11 | 4.15 | 4.37 | 224.82 | 85.19 | 109 | 108.12 | 124.61 | 51.19 | 50.89 | 42.79 | 48.34 | 55.12 | 44.45 | 4.28 | 4.71 | 6.38 | 6.17 | 4.33 | 5.37 |
| SexeOBP19 | 0.32 | 0.37 | 0.34 | 0.32 | 0.29 | 0.35 | 0 | 0.71 | 0 | 0.8 | 0 | 0 | 0 | 0 | 0.34 | 0 | 0 | 0 | 0.41 | 0.39 | 0 | 0.87 | 0 |
| SexeOBP20 | 0.1 | 0 | 0.1 | 0 | 0 | 0 | 0 | 0 | 0 | 0 | 0 | 9.38 | 7.41 | 4.3 | 7.48 | 7.18 | 5.19 | 9.61 | 3.62 | 0.73 | 9.48 | 7.99 | 3.48 |
| SexeOBP21 | 0.05 | 0.32 | 1.28 | 3.02 | 0.69 | 0.25 | 0 | 0 | 0.13 | 0 | 0 | 0.06 | 0.13 | 0.06 | 0.12 | 0.74 | 0.07 | 0.15 | 0 | 0 | 0 | 0 | 0 |
| SexeOBP22 | 0 | 0 | 0.16 | 0.16 | 0 | 0.09 | 0 | 0 | 0 | 0 | 0 | 0.18 | 0.37 | 0 | 0.08 | 0.85 | 0.56 | 0.21 | 0 | 0 | 0.29 | 0.42 | 0.2 |
| SexeOBP23 | 0 | 0 | 0.17 | 0 | 0.15 | 0.17 | 0 | 0 | 0 | 0 | 0 | 0 | 0 | 0 | 0 | 0 | 0 | 0 | 0 | 0 | 0 | 0 | 0 |
| SexeOBP24 | 0 | 0.12 | 0.33 | 0.32 | 0.6 | 0 | 0 | 0 | 0 | 0 | 0 | 4.29 | 3.72 | 4.49 | 4.23 | 2.69 | 2.02 | 2.43 | 3.21 | 2.09 | 1.19 | 1.14 | 2.21 |
| SexeOBP25 | 0 | 1.23 | 0 | 0 | 0 | 0 | 0 | 1.21 | 1.32 | 0 | 0 | 0 | 0 | 0 | 0 | 0 | 0 | 0 | 0 | 0 | 0 | 0 | 0 |
| SexeOBP26 | 1.02 | 0.51 | 1.2 | 0.09 | 0.5 | 0.29 | 0 | 0.1 | 0 | 0.11 | 0.11 | 172.24 | 161.82 | 133.17 | 203.54 | 152.03 | 121.75 | 84.44 | 75.04 | 64.48 | 112.27 | 97.34 | 79.97 |
| SexeOBP27 | 0.53 | 0.62 | 0.76 | 0.92 | 0.17 | 0.2 | 0 | 0 | 0 | 0 | 0 | 0 | 0 | 0 | 0 | 0 | 0 | 0 | 0 | 0 | 0 | 0 | 0 |
| SexeOBP28 | 0.17 | 0 | 0 | 0.08 | 0.14 | 0.04 | 0.13 | 0.16 | 1.83 | 0.8 | 0.49 | 0.64 | 1.4 | 0.46 | 0.73 | 0.66 | 0.31 | 0.15 | 0.19 | 0.05 | 0.36 | 0.39 | 0.52 |
| SexeOBP29 | 0.01 | 0.04 | 0 | 0.03 | 0.06 | 0 | 0 | 0.07 | 0 | 0 | 0.12 | 135.28 | 141.46 | 133.6 | 104.1 | 117.56 | 82.71 | 0.37 | 0.01 | 0 | 0.09 | 0.05 | 0.04 |
| SexeOBP30 | 20.34 | 14.57 | 19.38 | 0.44 | 8.01 | 6.7 | 0.32 | 38.21 | 0.38 | 0.4 | 36.51 | 93.37 | 128.57 | 149.58 | 107.35 | 92.32 | 96.3 | 1.06 | 0.86 | 0.51 | 0.5 | 0.59 | 0.58 |
| SexeOBP31 | 0 | 0 | 0 | 0 | 0 | 0 | 0 | 0 | 0 | 0 | 0 | 13.6 | 14.27 | 12.51 | 12.21 | 14 | 10.76 | 0 | 0 | 0 | 0 | 0 | 0 |
| SexeOBP32 | 0.17 | 0.1 | 0.35 | 0.25 | 0.29 | 0.39 | 0 | 0.09 | 0.11 | 0.6 | 0 | 0 | 0.21 | 0.37 | 0.09 | 0 | 0.1 | 0.12 | 0.11 | 0 | 0.11 | 0.12 | 0.11 |
| SexeOBP33 | 0 | 0 | 0.24 | 0.16 | 1.13 | 0 | 0 | 0 | 0 | 0 | 0 | 0.36 | 0 | 0 | 0 | 0 | 0.37 | 0 | 0 | 0.57 | 0 | 0.85 | 0.24 |
| SexeOBP34 | 0 | 0 | 0 | 0.58 | 0 | 0 | 0 | 0 | 0 | 0 | 0 | 0 | 0 | 0 | 0.3 | 0 | 0 | 1.21 | 0 | 0 | 0.35 | 0 | 0 |
| SexeOBP35 | 0 | 0 | 0 | 0 | 0 | 0.03 | 0.1 | 0 | 0.07 | 0 | 0.15 | 112.51 | 109.19 | 106.17 | 96.42 | 148.62 | 105.27 | 0.13 | 0.14 | 0.08 | 0 | 0 | 0.04 |
| SexeOBP36 | 0 | 0.12 | 0 | 0 | 0 | 0 | 0 | 0 | 0.06 | 0 | 0 | 0.25 | 0.8 | 1.69 | 0.73 | 2.24 | 1.53 | 0.07 | 0 | 0.2 | 0 | 0 | 0 |
| SexeOBP37 | 49.68 | 68.35 | 43.48 | 71.9 | 47.28 | 45.06 | 0.07 | 0.14 | 0 | 0.04 | 0.06 | 4.82 | 5.31 | 5.01 | 6.74 | 4.11 | 3.26 | 4.98 | 4.32 | 5.24 | 5.85 | 6.97 | 5.57 |
| SexeOBP38 | 0.82 | 0.19 | 0 | 0 | 0 | 0.18 | 0 | 0 | 0 | 0 | 0 | 0.19 | 0 | 0 | 0 | 0 | 0 | 0.89 | 1.89 | 1.34 | 0.19 | 0.63 | 0.41 |
| SexeOBP39 | 0 | 0 | 0 | 0 | 0 | 0 | 0 | 0 | 0 | 0 | 0 | 0 | 0 | 0 | 0 | 0 | 0 | 0 | 0 | 0 | 0 | 0 | 0 |
| SexeOBP40 | 0 | 0 | 0 | 0 | 0 | 2.65 | 0 | 2.94 | 0 | 0 | 2.68 | 0 | 3.08 | 0 | 0 | 0 | 0 | 0 | 0 | 0 | 3.02 | 0 | 3.21 |
