# Supplementary material for: Sex- and stage-dependent expression patterns of odorant-binding and chemosensory protein genes in Spodoptera exempta
Source: PeerJ. 2021 Sep 13;9:e12132. doi: 10.7717/peerj.12132 (PMC8445084; doi:10.7717/peerj.12132)
Supplement: Supplemental Information 11 — L = larvae, P = pupae, M = male adults, F = female adults. [file peerj-09-12132-s011.docx]

Table S8 The TPM values of *S. exempta* CSPs in different samples. L = larvae, P = pupae, M = male adults, F = female adults.

| Gene name | L1 | L2 | L3 | L4 | L5 | L6 | P1 | P2 | P3 | P4 | P5 | M1 | M2 | M3 | M4 | M5 | M6 | F1 | F2 | F3 | F4 | F5 | F6 |
| --- | --- | --- | --- | --- | --- | --- | --- | --- | --- | --- | --- | --- | --- | --- | --- | --- | --- | --- | --- | --- | --- | --- | --- |
| SexeCSP1 | 0.42 | 1.46 | 0.45 | 0.21 | 0 | 0.46 | 0 | 0.46 | 0.26 | 1.57 | 0.25 | 0.24 | 0.25 | 0 | 0 | 0.26 | 0.5 | 0 | 0 | 0 | 0 | 0 | 0 |
| SexeCSP2 | 17.56 | 24.35 | 33.29 | 24.85 | 31.06 | 28.61 | 0.07 | 0.67 | 0.53 | 0.42 | 1.16 | 241.92 | 217.13 | 202.6 | 333.69 | 303.56 | 284.83 | 82.85 | 81.32 | 90.59 | 117.73 | 117.67 | 138.41 |
| SexeCSP3 | 0 | 0.24 | 0 | 1.05 | 0.19 | 0 | 0.23 | 0 | 0.25 | 0 | 0 | 0 | 0 | 0.22 | 0.43 | 0 | 0.24 | 0 | 0 | 0 | 0 | 0 | 0 |
| SexeCSP4 | 387.78 | 254.48 | 829.05 | 426.87 | 615.57 | 558.03 | 0.08 | 0.15 | 0.52 | 0.71 | 0.11 | 21.74 | 21.1 | 12.35 | 5.98 | 4.48 | 4.2 | 6.05 | 9.11 | 5.14 | 3.83 | 42.42 | 5.72 |
| SexeCSP5 | 0.24 | 0 | 0.31 | 6.84 | 0.18 | 0.28 | 0 | 1.26 | 0.3 | 0.92 | 0.55 | 11.47 | 10.03 | 6.51 | 13.93 | 8.84 | 6.4 | 2.24 | 1.46 | 0.24 | 5.34 | 4.33 | 2.03 |
| SexeCSP6 | 0.06 | 0.31 | 0.14 | 1.04 | 0.51 | 0.22 | 4.5 | 4.95 | 7.35 | 7.11 | 6.44 | 0.86 | 0.71 | 0.57 | 1.71 | 0.57 | 0.24 | 0.73 | 0.34 | 0.34 | 0.51 | 0.18 | 0.53 |
| SexeCSP7 | 0.56 | 0.22 | 1.59 | 0.19 | 0.35 | 0.2 | 1.28 | 0.21 | 0 | 0.23 | 0.22 | 0 | 0.67 | 0.2 | 0.2 | 0.23 | 0.45 | 0.5 | 0 | 0.69 | 0.7 | 1.53 | 0.25 |
| SexeCSP8 | 19.54 | 21.98 | 6.36 | 13.17 | 13.47 | 32.55 | 2.06 | 1.17 | 2.43 | 3.16 | 0.17 | 1.08 | 0.88 | 0.64 | 0.76 | 0.17 | 0.77 | 0.65 | 0 | 0.29 | 1.07 | 0.68 | 1.33 |
| SexeCSP9 | 284.64 | 282.55 | 252.09 | 339.17 | 474.13 | 331.82 | 10.88 | 28.69 | 16.49 | 27.57 | 19.17 | 358.56 | 315.15 | 323.66 | 388.34 | 341.17 | 342.4 | 186.59 | 178.87 | 184.57 | 225.84 | 240.78 | 276.54 |
| SexeCSP10 | 0 | 7.27 | 6.79 | 6.39 | 26.02 | 0 | 14.76 | 22.16 | 65.59 | 123.16 | 0 | 49.53 | 23.05 | 40.46 | 83.95 | 40.81 | 69.06 | 92.8 | 32.59 | 43.34 | 38.53 | 24.58 | 62.2 |
| SexeCSP11 | 4.5 | 3.06 | 1.9 | 1.75 | 1.52 | 1.87 | 0.97 | 7.01 | 5.44 | 7.8 | 2.89 | 1.94 | 4.29 | 3.75 | 2.77 | 3.26 | 4.95 | 4.51 | 2.2 | 1.02 | 5.35 | 4.71 | 2.32 |
| SexeCSP12 | 11.31 | 10.41 | 4.84 | 12.22 | 15.83 | 8.48 | 28.08 | 29.09 | 41.13 | 38.87 | 41.79 | 47.92 | 47.31 | 39.52 | 40.84 | 31.87 | 37.17 | 21.99 | 20.87 | 25.19 | 31.78 | 27.96 | 26.13 |
| SexeCSP13 | 7.61 | 11.35 | 3.37 | 4.62 | 8.9 | 13.16 | 0.54 | 0.9 | 0 | 0.29 | 0.71 | 7.01 | 11.04 | 7.32 | 9.76 | 6.78 | 4.39 | 5.12 | 4.66 | 4.24 | 9.78 | 9.47 | 4.52 |
| SexeCSP14 | 0.27 | 0.16 | 0 | 0 | 0.65 | 0.77 | 0.48 | 0.92 | 0.85 | 0.87 | 0.5 | 0.32 | 0.33 | 0.15 | 0.59 | 0.68 | 0.17 | 0.75 | 0.89 | 0.17 | 1.4 | 0.95 | 0.74 |
| SexeCSP15 | 0.21 | 0.34 | 0.46 | 0.52 | 0.62 | 0.4 | 0.34 | 0.24 | 0.26 | 0.27 | 0.35 | 0.34 | 0.77 | 0.23 | 0.39 | 0.44 | 0.53 | 0.4 | 0.19 | 0.27 | 0.36 | 0.39 | 0.38 |
| SexeCSP16 | 0 | 0 | 0 | 1.39 | 0 | 0 | 0 | 0 | 0 | 0 | 0 | 0 | 0 | 0 | 0 | 0 | 0 | 0 | 0 | 0 | 0 | 0 | 0.92 |
| SexeCSP17 | 42.64 | 39.27 | 34.64 | 52.92 | 63.63 | 53.1 | 167.36 | 542.5 | 466.16 | 447.93 | 761.26 | 31.63 | 24.98 | 27.62 | 33.88 | 24.08 | 36.17 | 12.69 | 12.04 | 12.59 | 23.52 | 19.83 | 21.36 |
| SexeCSP18 | 0 | 0 | 1.53 | 0.36 | 0.32 | 0 | 0.4 | 0.4 | 0.44 | 0.45 | 0 | 0 | 0 | 0 | 0.38 | 0 | 0 | 0 | 0 | 0 | 0.44 | 0 | 0 |
| SexeCSP19 | 97.05 | 60.42 | 75.78 | 213.24 | 144.52 | 112.27 | 27.77 | 20.56 | 114.92 | 199.18 | 48.63 | 34.9 | 31.64 | 29.17 | 47.85 | 30.56 | 32.85 | 24.63 | 22.45 | 22.31 | 17.96 | 20.34 | 22.97 |
| SexeCSP20 | 0.02 | 0.08 | 0 | 0.06 | 0 | 0.03 | 13.47 | 1.91 | 4.93 | 5.3 | 6 | 0.71 | 0.85 | 0.48 | 1.26 | 1.02 | 1.03 | 0.72 | 0.32 | 0.41 | 0.52 | 0.57 | 0.49 |
| SexeCSP21 | 0.13 | 0.37 | 0.19 | 0.09 | 0.09 | 0.1 | 0.53 | 0 | 0.05 | 0.11 | 0.22 | 0.16 | 0.16 | 0.05 | 0.1 | 0.17 | 0.39 | 0.06 | 0.06 | 0.52 | 0 | 0.31 | 0.47 |
| SexeCSP22 | 0.3 | 0.53 | 0.32 | 0 | 0.14 | 0 | 0.17 | 0.17 | 0 | 0 | 0.36 | 0.53 | 0 | 0.16 | 0.64 | 0.37 | 0.18 | 0 | 0.39 | 0.37 | 0.38 | 0 | 0.4 |
| SexeCSP23 | 86.31 | 85.28 | 142.5 | 89.01 | 92.02 | 88.38 | 0 | 0.1 | 0 | 0 | 0.12 | 0.32 | 0.33 | 0.2 | 0.43 | 0.97 | 0.24 | 0.25 | 0.35 | 0.12 | 0 | 0.14 | 0 |
| SexeCSP24 | 0 | 0 | 1.35 | 0 | 0.03 | 0 | 0.11 | 1.28 | 0 | 0 | 1.5 | 4.25 | 5.97 | 5.11 | 3.4 | 4.47 | 1.32 | 0 | 0 | 0.36 | 0.07 | 0.5 | 0.1 |
| SexeCSP25 | 5.92 | 6.57 | 0.67 | 6.49 | 4.49 | 1.32 | 2.43 | 12.56 | 4.73 | 7.48 | 10.96 | 1307.26 | 1288.78 | 1150.45 | 1585.01 | 1229.1 | 1083.86 | 291.95 | 265.18 | 384.68 | 480.75 | 440.07 | 413.75 |
| SexeCSP26 | 326.57 | 325.52 | 233.15 | 365.51 | 556.43 | 389.6 | 4372.22 | 2126.33 | 2768.23 | 2042.62 | 3262.85 | 319.57 | 392.22 | 383.72 | 579.28 | 391.73 | 424.38 | 218.63 | 147.49 | 205.98 | 334.04 | 316.01 | 280.15 |
| SexeCSP27 | 0.31 | 0.25 | 0.11 | 0.22 | 0.2 | 0.47 | 0.12 | 0.23 | 0.38 | 0.26 | 0 | 0.25 | 0.13 | 0.34 | 0 | 0 | 0.38 | 0.14 | 0.27 | 0 | 0 | 0.43 | 0.28 |
| SexeCSP28 | 126.72 | 98.16 | 105.2 | 287.18 | 186.84 | 97.4 | 143.24 | 116.5 | 462.54 | 897.49 | 64.68 | 158.23 | 88.47 | 187.5 | 59.98 | 85 | 59.79 | 13.41 | 14.77 | 8.81 | 4.72 | 5.99 | 4.69 |
| SexeCSP29 | 0 | 0.18 | 0.22 | 0.11 | 0.1 | 0.06 | 0.12 | 0 | 0 | 0.13 | 0.13 | 0.12 | 0.06 | 0.11 | 0.11 | 0.13 | 0.06 | 0.15 | 0.2 | 0.13 | 0.13 | 0.14 | 0.14 |
| SexeCSP30 | 120.58 | 140.27 | 67.14 | 211.04 | 338.27 | 177.17 | 674.28 | 714.53 | 688.88 | 1018.84 | 656.73 | 1286.65 | 1301.86 | 1552.31 | 2047.67 | 1289.1 | 1358.27 | 1248.59 | 866.95 | 998.43 | 1197.64 | 1359.91 | 1138.92 |
| SexeCSP31 | 0 | 1.66 | 0 | 0 | 0 | 0 | 0 | 0 | 0 | 0 | 0.79 | 0 | 0 | 0 | 0 | 0 | 0 | 0 | 0 | 0 | 0 | 0 | 0 |
| SexeCSP32 | 0.37 | 0 | 0 | 0.37 | 0 | 0 | 0.82 | 0.41 | 0 | 0.46 | 0.42 | 0 | 0 | 0 | 0 | 0 | 0.43 | 0 | 0 | 0 | 0 | 0 | 0 |
| SexeCSP33 | 352.43 | 747.84 | 277.1 | 302.44 | 239.98 | 383.52 | 6.97 | 16.19 | 18.97 | 50.88 | 11.63 | 0.1 | 0.11 | 0.09 | 0 | 0 | 0.1 | 0 | 0 | 0.64 | 0.33 | 0.35 | 0.22 |
