# Supplementary material for: Sex- and stage-dependent expression patterns of odorant-binding and chemosensory protein genes in Spodoptera exempta
Source: PeerJ. 2021 Sep 13;9:e12132. doi: 10.7717/peerj.12132 (PMC8445084; doi:10.7717/peerj.12132)
Supplement: Supplemental Information 14 [file peerj-09-12132-s014.pdf]

```
                     *        20         *        40         *        60         *        80         *       100         *       120
SexeOBP1  : ---------------------MNNLFVVYLVVLC------------------------A--------------------------------------------------------------- :  14
SexeOBP2  : --------------------MQKETNMLLAKIVK-----------------------LL--------------------------------------------------------------- :  16
SexeOBP3  : ----------------------MKLFLLFCTVFV----------------------TNG--------------------------------------------------------------- :  15
SexeOBP4  : ----------------MKTCTFQFVCCILSIFLL------------------------F--------------------------------------------------------------- :  19
SexeOBP5  : --------------------MFKLSVLVCCLYFC---------------------ALTP--------------------------------------------------------------- :  18
SexeOBP6  : ---------------------MSFSSIIWCAVIC------------------------I--------------------------------------------------------------- :  14
SexeOBP7  : ----------------------MIFRFLLCFYLV---------------------------------------------------------------------------------------- :  12
SexeOBP8  : ------------------MLNSVFFLFTFGVLFL---------------------------------------------------------------------------------------- :  16
SexeOBP9  : ------------------MTKVLFAIVLTMVTFG----------------------VTL--------------------------------------------------------------- :  19
SexeOBP10 : ------------------MLRINVAVAVFIVNLI---------------------------------------------------------------------------------------- :  16
SexeOBP11 : -------------------MDRKRTCLLVIAMFL---------------------------------------------------------------------------------------- :  15
SexeOBP12 : ------------------MLRVGVAVAVFFANLV---------------------------------------------------------------------------------------- :  16
SexeOBP13 : --------------------MSKFTCIVFCIVAA----------------------SLT--------------------------------------------------------------- :  17
SexeOBP14 : --------------------MSKLSCIVVCALAM-----------------------RL--------------------------------------------------------------- :  16
SexeOBP15 : --------------------MYKFACLILCVVAV-----------------------SL--------------------------------------------------------------- :  16
SexeOBP16 : ---------MTFEGQIAQYNMAKFISLVFSLVIV-----------------------SL--------------------------------------------------------------- :  27
SexeOBP17 : -----------------MSTCTDLTRIGYVMLFW----------------------FLV--------------------------------------------------------------- :  20
SexeOBP18 : --------------------MKTLLVFAACVLLA---------------------------------------------------------------------------------------- :  14
SexeOBP19 : ----------------------MRSFVVFCIVLV---------------------------------------------------------------------------------------- :  12
SexeOBP20 : ------------MARRQRGAMFTETLPLFVILVA---------------------------------------------------------------------------------------- :  22
SexeOBP21 : ---------------------MFHSGVVYFLVIA----------------------VFL--------------------------------------------------------------- :  16
SexeOBP22 : ---------------MYSINYFLFSIILFVMFNN---------------------------------------------------------------------------------------- :  19
SexeOBP23 : ----------------------MEKILIFTFITL------------------------S--------------------------------------------------------------- :  13
SexeOBP24 : --------------------MAKVIYIVFFVVAV----------------------SLC--------------------------------------------------------------- :  17
SexeOBP25 : --------------------MSKFTCLVLCVVAV-----------------------SL--------------------------------------------------------------- :  16
SexeOBP26 : ---------------MSVMRRSSFLVVLFCFVSV---------------------------------------------------------------------------------------- :  19
SexeOBP27 : ----------------------MWNCLVVFLAIC---------------------------------------------------------------------------------------- :  12
SexeOBP28 : ------------------MCLVKYHVLVLCVLLVGGYALNCRSSGGPKEAELKNIYKKCLKMQEGKNSSKGNSAQDWKEPRIQIQRNDWDRGHSGSKENKNSRDDSRMGNKDKKGGSGMRDS : 104
SexeOBP30 : -------MAAECEARASGLASAAAGAAGCCVAACATAPAPGPARPDPHMTCSRALALLA--------------------------------------------------------------- :  52
SexeOBP31 : MKYKARRTLTQREKEKMSNQKTNVLALILLILFS--------------------FAFYL--------------------------------------------------------------- :  39
SexeOBP32 : --------MMGIDAIHDNNVKIDKDTIITRNLKL-------------EKRSRGQKSDSN--------------------------------------------------------------- :  38
SexeOBP33 : -----------------MVRPQKPNKSMFVIFFV---------------------------------------------------------------------------------------- :  17
SexeOBP34 : ----------------MITHPSPMTSTLQLKHTI---------------------------------------------------------------------------------------- :  18
SexeOBP35 : -------------------------------------------------------------------------------------------------------------------------- :   -
SexeOBP36 : -----------------MLLIKVFSCILPFVVGL-------------------RIKRSS--------------------------------------------------------------- :  23
```

```
                   *        140        *        160        *        180        *        200        *        220        *        240
SexeOBP1  : ----------------------------------------------------------------------------------------GCSYGMT-----------RAQVKKTMGVVKNQCM :  37
SexeOBP2  : ----------------------------------------------------------------------------------------IVVATCEAMT--------MKQIRNTGKMMRKTCQ :  42
SexeOBP3  : ----------------------------------------------------------------------------------------IDAYVYKQEAEMKQ---ILNKV-PTINTYFTECI :  45
SexeOBP4  : ----------------------------------------------------------------------------------------GQSYGMT-----------RQQLKNSGKLMKKSCM :  42
SexeOBP5  : ----------------------------------------------------------------------------------------YLASAMTAEQ--------KAQIHEHFEMVGKYCN :  44
SexeOBP6  : ----------------------------------------------------------------------------------------STVYATTKFR---------------NKEGIEKCF :  33
SexeOBP7  : -----------------------------------------------------------------------------------------ELYGAHART--------DEEIKAWFFREGIDCN :  37
SexeOBP8  : -----------------------------------------------------------------------------------------SVEALS-----------MEDLKQRYVDNILECS :  38
SexeOBP9  : ----------------------------------------------------------------------------------------SASTKEAKTTTMSD---QVNSIDVDVLAVMDMCN :  50
SexeOBP10 : ----------------------------------------------------------------------------------------SGWGQTTQDP--------RVGEMLLAKRYQMECI :  42
SexeOBP11 : ------------------------------------------------------------------------------------------ASGSDAMS--------RQQLKNSGKMLKKNCM :  39
SexeOBP12 : ----------------------------------------------------------------------------------------CGAQDSKDES--------LNEIVMLSKRYQLECI :  42
SexeOBP13 : ----------------------------------------------------------------------------------------KVSHAVTEEE--------KEAFRAALEPVIVKCS :  43
SexeOBP14 : ----------------------------------------------------------------------------------------CCDFVASEDA--------NSVFHDVIRPMIFECA :  42
SexeOBP15 : ----------------------------------------------------------------------------------------SGVHATAEE---------KAAFIEAVKPHIQECS :  41
SexeOBP16 : ----------------------------------------------------------------------------------------NAVYAEED----------VESSSKEDHSIMDECA :  51
SexeOBP17 : ----------------------------------------------------------------------------------------KNVAALSDRE--------KSAIQKELTSVGLQCI :  46
SexeOBP18 : -------------------------------------------------------------------------------------------QALTDEQ------------KEKLKKHRSECL :  33
SexeOBP19 : ----------------------------------------------------------------------------------------AGVCAT-----------------EKGNKITSECI :  29
SexeOBP20 : -----------------------------------------------------------------------------------------VTHGGKDKPVF------SDEIKEIIQTVHDECV :  49
SexeOBP21 : ----------------------------------------------------------------------------------------KNASAITDEL--------KAHIEAKFLTVGAECI :  42
SexeOBP22 : ----------------------------------------------------------------------------------------CFVHSMT-----------REQIKNSGKLIKKTCS :  42
SexeOBP23 : ----------------------------------------------------------------------------------------GFAHARISV---------MYAHDKLSDLVAQQCL :  38
SexeOBP24 : -----------------------------------------------------------------------------------------SIQADDDDD--------NSELIKNINQKLLDCT :  42
SexeOBP25 : ----------------------------------------------------------------------------------------SGVHATAEE---------KAAFIEAVKPYVQECS :  41
SexeOBP26 : -------------------------------------------------------------------------------------------HAQSADD--------ESAIRDALRPYVLECA :  42
SexeOBP27 : ----------------------------------------------------------------------------------------CCVYGLT-----------EEELKSEFTKLIIKCN :  35
SexeOBP28 : RDDMMGRRDDMMTRGDDRSDNRKHRTDDRMGNGNDRSDNRGRMGNKNNRNDMNGERDNRYGRDDYFNGREDFPQSGEYGSDIGQYNNNYYSTTQSTRRYKRERRPSNSGQRSQYNPNSHKIS : 226
SexeOBP30 : ----------------------------------------------------------------------------------------LVAVSQQATTGCKNCIMLGKEEKAMFRAHSDACV :  86
SexeOBP31 : ----------------------------------------------------------------------------------------TISFTPLTKEEQMERYNKMSEDIEPFRKNLTECA :  73
SexeOBP32 : ----------------------------------------------------------------------------------------KNVDEKEPDWSYASFPKEISEHVENFKKNMSECL :  72
SexeOBP33 : ---------------------------------------------------------------------------------------------VESKF--------GEIVKRTVIATAHTCM :  38
SexeOBP34 : ------------------------------------------------------------------------------------------------------------LFQEKGNKIASECI :  32
SexeOBP35 : ---------------------------------------------------------------------------------------------------------------------MTECL :   5
SexeOBP36 : ----------------------------------------------------------------------------------------GTMVDFTDPK--------VQGHLDALVRMAQSCV :  49
                                                                                                                                    c
```

```
                 *         260         *         280         *         300         *         320         *         340         *         360
SexeOBP1  : PKNSV-TEEQVGRIEQGVFI---EDRNVMCYVA-CIYKTMQVV-KNDK-LDMSLILKQVDIL---YPPEL--KEPVKKSIAACIHSQ-----DNYSDLCEGVFYASKCLYEKDPNS------ : 136
SexeOBP2  : PKNNA-EDEKIDPIADGVFI---DEKEVKCYMA-CIMKMANTI-KNGK-LNYDAAIKQADLL---LPDDI--KEPAKEAITACRKVA-----DAHKDICDASFHITKCIYNHNPGIFYFP-- : 145
SexeOBP3  : VETNE-DALQDETLLLEE-----GVRNADELIN-CFFNKAGIIDDGGR-LSNGAARSILNLI---GVEDDE----ADQLIGKCTS-------IAGIDGSDMAFAIFKCFEDGVKK------- : 138
SexeOBP4  : PKNDV-TEEEIGDIEKGKFI---ENRNVMCYIA-CVYTMTQVV-KNNK-LSYEAVIKQVDVM---FPAEM--RDAVKAAATHCKETT-----KKYKDLCESAYWTAKCMYDYDAENFVFP-- : 145
SexeOBP5  : KDSTMITADDIVNLRAKKVP---TGPNAPCFLA-CVMKQIGVMDDSGM-LQKETALEMAKAV---FQDAEE-LKAIEDYLHSCSHINGESV-SDGAAGCDRAMLSYKCMTENASKFGFDI-- : 154
SexeOBP6  : EKFGV-SMTEKESSEKEDV----SSVDP-CFWA-CSFKTIGFLNSEGQ-YNPEITHSHFKTEELSFLGTAR-LDKLKDLIAKCDAALEKITGTDEKAECDRGLQLAKCYVEDVKDIFLDDSS : 146
SexeOBP7  : NEHPI-SPTEMLSLKENKIP---NTNNAKCFVA-CVFKKTGMLDSKGM-FDADHSIAMIQKD---FADDPQKLESSKKLLETCKKVNDEAV-SDGEKGCERSVLLHKCFVETAPELGIKLP- : 148
SexeOBP8  : KQYPI-DRADAEQLQNRVMP---DKDSAKCLFA-CVYKLAGVMNDQGE-MSVEGVNALSRKY---LAGDPEKLTKSEQFTEACKTVNDAPV-SDGTRGCDRAALIFKCTVEKSPEFNFV--- : 147
SexeOBP9  : DSYRI-DPSYIQALNESGSFIDETDKTPKCFIR-CVFENVGIVSEDGMQFNPARAAVIFAGE---RNGKP--MEDIADMTALCATD------RQETCPCDRSYKFLRCLMSMEIERYEKS-- : 157
SexeOBP10 : EETRV-DPDIIAGIKNGRWSIPWTARPLAMKWALCVMMKRGLMTKEGV-YKL-------------------------------------------------------------------- :  92
SexeOBP11 : NKIGV-TEDQVGSIDKGKFI---EDRKVMCYIA-CIYELTNVI-KNNK-LSYEASIKQIDLM---YPPDV--KESAKAAVEKCKDVQ-----KKYKDICEVSFYAAKCMYEYKPEDFIFA-- : 142
SexeOBP12 : EETRV-DADIIATIKTGGWNIPSHDTFTVRQWALCVMMKRGLMSKEGV-YKLDVALKM-------VPKED--RDAAEKLIDSCLS-------QKALPADEIAFLFIKCFQRTRSKFPVSIFI : 146
SexeOBP13 : EEHGV-SEADIEAAKEAGSA---DAIKP-CFLG-CVMKKIEVLDSKGL-YDPETGLGKLRKF---VKDEDE-YAKFEEIAKKCLKVNDESV-SDAEAGCDRAKLLLGCFLEHKVEMPF---- : 149
SexeOBP14 : EQYNL-TEEDLKKNRGPDAL----KKYPPCFVG-CVLKKFNIINDKGQ-YDADAGLTTIKTL---LPNKEY-FEKISGVFKECTSVNDKTV-SDGDAGCERAVLATTCYMEHKTAIIA---- : 148
SexeOBP15 : KEHGV-TPEDIKSAKEAGSA---DNINS-CFLS-CVYKKAEVVSIN-------------------------------------------------------------------------- :  81
SexeOBP16 : NKFGI-TLDEITVAFRSGNV---GALDP-CFWK-CCYGGTGLINKEGL-YDLDATLSFIKTK---FHDED--YTQFQETARMCEKVNKEDV-KDGEAGCERAAMLVSCFLKGRGDELLQ--- : 157
SexeOBP17 : QQHPL-SLSDIRAFRNKMIP---DGKKPKCFVA-CLFKKIGVMDDMGM-ISPMKAQENAKKV---FKDNQEHIKNVNEIMEKCSAVNQQNT-MDGNKGCDRAKLAFNCLTENADKYGFDFDF : 158
SexeOBP18 : TETKV-DEQLVNKLKGGDYK---TDSEPLKKYALCMMMKSELMTKDGK-FKKDVALAK-------VPNPAD-KPTVEKLIDACLA-------NKGNTPHQTAWNYVKCYHEKDPKHAIFL-- : 133
SexeOBP19 : KESGV-KSEVLAEAKKGNIS---DDPAFKAFTF-CFFKKAGIVGEDGK-LNRDVALAK-------LPSGVD-KSEAEKLLDSCKS-------KTGKDAVDTVFEIFKCYQHGTKSH------ : 124
SexeOBP20 : AKTGV-AEEDITNCENGIFK---EDTKLKCYMF-CLLEEASLVDDDDT-VDYDMLVSL-------IPDEY--YERTTKMIFACKHLD-----SPDKDRCQRAFEVHKCSYEKDPDLYFLF-- : 149
SexeOBP21 : KEHPL-TIEDLAAFKNRVFP---DGENAGCFSA-CIFNKLGLFDDKGT-LSHLTALENAKKV---FEDEEE-LGSIEKFLTSCAKVNDEEV-SDGEKGCERAKLAYNCFIENYEQLGFDLEF : 153
SexeOBP22 : AKHDL-TEDEVKDVDKGKFL---ETKDFMCYIA-CVYKMGQSV-KGST-INHDMMLRQVDMM---FPNDM--KAPVKAAIEHCRPVA-----KNYKDLCEASYWTAKCIYDFDSANFMFP-- : 145
SexeOBP23 : --------SEMYPKNKRIEI---QESDEPCIIF-CVLKKFGIISASGV-INLDIYRKRVQ-----IAHQLDQKTSIMDYGGSCMENAEAT--QHKQDVCKKAKVFNDCTHLYRILLM----- : 135
SexeOBP24 : KKFDI-SVEQFHETIESGDA---YAVAP-CFWS-CCFKKVGVINSEGQ-YDLDATLDLSKKI---FSTWE--YENVEDIVKKCEKVNELV--SDGNTGCDRSISLAACIFENWKKINPNKFS : 150
SexeOBP25 : KEHGV-TPEDIKSAKAAGNA---DGINS-CFLS-CVYKKAEVINEKGE-YDV-------------------------------------------------------------------- :  86
SexeOBP26 : EEFGI-TEEEFEEAKKKASA---ADIDP-CFMS-CFLKKAQFFDDQGK-FDVDSTMAFAKEN---LSSEPM-MKFVEAVGDECGKS---------------------------------- : 117
SexeOBP27 : KDGQV-DMTELVKLQNYVVP---TKQTTKCVLA-CAYKAAEVMNAKGE-YDIEHAYKVAEKM---KNGDEKRLVNAKKMADLCVKVNEESV-SDGDKGCDRAAMIFKCTVENAPKFGFKL-- : 145
SexeOBP28 : GYEDTFRSDERNTTENNSSK---ETDNKSCALH-CFLENLEMTGEDGM-PDRYLVTHAITKD---VKNEDL-RDFLQESIEECFQILDN---ENTEDKCEFSKNLLICLSEKGRANCDDWKD : 336
SexeOBP30 : AASRV-APRLVEAMLAGQLL---DDPALRRHVY-CVLLKCKLISKDGK-LQKAAVLGK-------MAARPD-AKNATKVLESCAD-------QTGDTPEDLAWNLFRCGYDKKALLFDYMPT : 187
SexeOBP31 : RQVKA-SAADVENFLKRIPQ---ASLQGKCFVA-CILKRTSII-KNNK-ISRKQLLEANRAV---YGEDSEVMSRLKIAVSECSKAV-----EGIFEICEYASVFNDCMHIKMEHILEQVTM : 180
SexeOBP32 : KEVQS-SDKRPVKRLSPKME---SPVHGECLIA-CVLKRNGVI-IHGK-VNKDNLIALVSKF---YSKDTRLMKKLEKNLDRCIEMS-----VRAQDDCTLASLLNDCTNDLMASNKHKL-- : 177
SexeOBP33 : DHVNA-TTKDLENLREEPP----YPETSACIVK-CLLEKIGVV-KGNR-YSKMGFMTAVTPL---VFTNKKKLEHMKTVSENCDKEVN----HKHETPCQLGNEVTTCIFKYAPELHFKS-- : 143
SexeOBP34 : KESGV-KSDVLAEAKKGNLG---DDPAFKEFTY-CFFKKVGIVGEDGK-LNRDVAIAK-------LPSGVD-KAEAEKLLDSCKS-------KTGKDAVETVYEIFKCYQHGTKSHIMFAS- : 132
SexeOBP35 : KEVQA-NDKRPVKRLSPKME---SPVHGECLIA-CVLKRNGVI-INGK-VNKENLITLVSKF---YSKDTRLMKKLEKNLDRCIEMS-----VRAQDDCTLASQLNDCTNDLMASNKQKIMV : 112
SexeOBP36 : IKVRA-TPKDVRAYFTNSSP---VSRSGQCFAA-CMLEQSDVI-NHGK-VNRELLVHLASLV---NGKQSRVVRKLNTVSRLCLDSI-----DGMSDRCQLASTYNDCLNENMIEFAFPLDI : 156
                                 c    C                                               c                        c
```

```
                 *       380         *
SexeOBP1  : -------------------------------- :   -
SexeOBP2  : -------------------------------- :   -
SexeOBP3  : -------------------------------- :   -
SexeOBP4  : -------------------------------- :   -
SexeOBP5  : -------------------------------- :   -
SexeOBP6  : K------------------------------- : 147
SexeOBP7  : -------------------------------- :   -
SexeOBP8  : -------------------------------- :   -
SexeOBP9  : -------------------------------- :   -
SexeOBP10 : -------------------------------- :   -
SexeOBP11 : -------------------------------- :   -
SexeOBP12 : -------------------------------- :   -
SexeOBP13 : -------------------------------- :   -
SexeOBP14 : -------------------------------- :   -
SexeOBP15 : -------------------------------- :   -
SexeOBP16 : -------------------------------- :   -
SexeOBP17 : -------------------------------- :   -
SexeOBP18 : -------------------------------- :   -
SexeOBP19 : -------------------------------- :   -
SexeOBP20 : -------------------------------- :   -
SexeOBP21 : -------------------------------- :   -
SexeOBP22 : -------------------------------- :   -
SexeOBP23 : -------------------------------- :   -
SexeOBP24 : DSA----------------------------- : 153
SexeOBP25 : -------------------------------- :   -
SexeOBP26 : -------------------------------- :   -
SexeOBP27 : -------------------------------- :   -
SexeOBP28 : -------------------------------- :   -
SexeOBP30 : NVATSESDNNS--------------------- : 198
SexeOBP31 : ERRMEAISKMTSDPDQWGEEEDELLKLVKDEL : 212
SexeOBP32 : -------------------------------- :   -
SexeOBP33 : -------------------------------- :   -
SexeOBP34 : -------------------------------- :   -
SexeOBP35 : NY------------------------------ : 114
SexeOBP36 : AEEAVRKMPFHLIQPNLPQEVRSMPY------ : 182
```
