# Supplementary material for: Sex- and stage-dependent expression patterns of odorant-binding and chemosensory protein genes in Spodoptera exempta
Source: PeerJ. 2021 Sep 13;9:e12132. doi: 10.7717/peerj.12132 (PMC8445084; doi:10.7717/peerj.12132)
Supplement: Supplemental Information 17 [file peerj-09-12132-s017.pdf]

```
                   *        20         *        40         *        60         *        80         *       100         *       120
SexeCSP1  : --MK--LVIILALVAVAVARPDDG--GT--YDSKYDNFNVDELVGNARLLKSY------------------------------AHCFLDDGK-CTPE-GNDFKKWIPEATTTSCGKCSEKQKA :  83
SexeCSP2  : --MNF-LVLSIVVSMAAFVAAET-------YTDRYDHINIDEIIENRKLLVPY------------------------------IKCTLDQGR-CTPE-GRELKGKIKCCLK------------ :  69
SexeCSP3  : --MK--LVIILALVAVALARPDDG--GF--YDTKYDNFNADELIENERLLKSY------------------------------AHCFLGDGK-CTPE-GNDFKKWIPEATTTSCGKCTEKQKA :  83
SexeCSP4  : --MKG-ILVLCLFVAAVSCKPET-------YDTRYDNFDVEALVGNVRLLTAY------------------------------GHCFLGTGP-CTPE-GSDFKKTIPDALRTGCAKCSPRQRH :  81
SexeCSP5  : MMTKS-FISMMLLIYLTIQSNATE--SST-YTTKYDGIDLDEILNNDRLLTGY------------------------------VNCLMDLGP-CTAD-GKELKKNLPDAIENDCKKCTERQRE :  87
SexeCSP6  : --MQV-IVVLVVVCMGLVAGLHVQ------AGPQMSDAQLEQILADRSTMQRH------------------------------IKCALAEGP-CDPV-GRRLRTLAPLVLRGACPQCSVQETR :  82
SexeCSP7  : --MKV-VFLVCVLAAVVYGEQ---------YTDKYDNIDLDEILHNEKILQSY------------------------------VNCCLDQGK-CTPD-GKELKSHIKEALENRCGKCTPAQKD :  79
SexeCSP8  : --MNA-LLIAVFAVAAPLAFG---------YDEKYDKLDVDKILGDDAVFTSY------------------------------INCMLDKGP-CSVEHSADFRQLLPEVISTACAKCNAIQRQ :  80
SexeCSP9  : -------------------------------------------------------------------------------------------------------MVDAIETGCSKCTEAQEQ :  18
SexeCSP10 : --MKS-MIVLCVLSVAALVVARPD--DSH-YTDRYDNVNLDEILSNRRLLVPY------------------------------VKCILDQGK------------------------------ :  56
SexeCSP11 : --MKV-ALLTLCFALGVLAQDM--------YENANDNFDISEVLGNERLLNSY------------------------------AKCLLNKGP-CTPE-VKQVKDKLPEALETRCAKCTDKKQ :  80
SexeCSP12 : --MRV-VLFICALVHLVVGQDVNDMVNMPKYDSRYDYLDVDAIFTNKRLVRNY------------------------------VDCLINSVR-CTPE-GKALKRILPEALRTKCVRCTERQKR :  88
SexeCSP13 : ---------------------------------------------------------------------------------MERGK-CSPE-GKELKEHLVDAIETGCSKCTEAQEK :  34
SexeCSP14 : --MKV-LVALSVFVVLAAAAPLTK--D---ELATLEAFDYDSLFADEEKRKVV------------------------------FDCLLDKGD-CGPY--KQIVDLSMKTILSNCAECSPSQKA :  82
SexeCSP15 : --MKC-IYVLSALLVFAAVQAEDK------YSTENDDLDIDAVVADVDALRGF------------------------------VGCFMDSVT-CHAV-AADFKKDLPEAVATSCSKCTEAQKH :  82
SexeCSP16 : MQIKY-ALVLCCVAAVSVAQT---------QRPPVSDTALDDALQDKRFIQRQ------------------------------LKCALGEGP-CDPI-GKRLKTLAPLVLRGACPQCSPQETK :  81
SexeCSP17 : --MRV-LVALSCLVVVAFAADK--------YNAKYDNFDVDTLITNDRLLKAY------------------------------INCFLEKGR-CTPE-GSDFKSEYYCYVDY----------- :  69
SexeCSP18 : --MKH-ILVALVVTIAVVKAQET-------YGTEYDNVNGEAIVSDDKQFQGF------------------------------VDCFTGAAP-CNEP-AAAFKRVLPEAIVQACGKCNPAQKH :  81
SexeCSP19 : --MKS-FIVVCLFGLAAIAMARPN--SST-YTDRYDNVNLDEILGNRRLLTPY------------------------------VKCILDEGK-CTAD-GKELKSHIREALEQNCAKCTDAQRR :  85
SexeCSP20 : --MRSWLLCLCVLTVVVSCYSQA---------NRYENFNPDAIVQNDRILLAY------------------------------YKCVMDKGP-CTRD-GKNFKRVLPETLATACGRCNPKQKT :  80
SexeCSP21 : --MKS-ILVLCLLVAAVSCRPES-------YDTRFDNFDVEALVGNVRLLTAY------------------------------GHCFLGNGP-CTPE-GSAFKKTIPDALRTGCGKCSPKQRH :  81
SexeCSP22 : --MKS-ILVLCLLVAAVSCKPET-------YDTRYDNFDVESLVGNVRLLTAY------------------------------GHCFLGNGP-CTPE-GSDFKKTIPDALRTGCGKCSPKQRH :  81
SexeCSP23 : --MKH-FLVLLIATIAVVTAQET-------YGTQYDNVNGEAIVSDDKQFQSF------------------------------VDCFRGDAP-CNEI-AAAFKRVLPEAIVEGCAKCNSVQKH :  81
SexeCSP24 : MWVQA-IPYFIVVHVVMGNKEVPK--ISREFSEGVQSMGFKVIYGDEDLTIINQVVSEAEKNSASKNKVHLNEAIKPLPAQDVKCLMSVDRYCSKE-MGQMKSVLIQAVKEDCAKCSVQQKD : 118
SexeCSP25 : --MKLIIAVVLCMVAVAWSRPAST------YTDKWDNINVDEILESQRLLKGY------------------------------VDCLLDRGR-CTPD-GKALKETLPDALEHECSKCTEKQKK :  83
SexeCSP26 : --MKV-VILVCVLAAVAYAHPHES--T---YTDKYDTIDLDEILSNRRLLVPY------------------------------VKCCLDEGK-CSPE-GKELKSHIKEALENKCGKCTKAQQN :  83
SexeCSP27 : --MKV-LVVLSVFLALAAAAPPKP--ITKEELAILEAFDYDAVFANEESRKLI------------------------------FDCLLDKGD-CGPY--KKVVELSKKSMYDECALCSPTQMA :  85
SexeCSP28 : --MKC-IYVLSVLLVFAGVLAEDK------YSTENDDLDIDAVVADLDSLKGF------------------------------VGCFMDSIT-CHAV-AADFKKDIPEAVSTHCAKCTDAQRH :  82
SexeCSP29 : ------------------------------------------------------------------------------------MDAVT-CHAV-AADFKKDLPEAVATSCSKCTDAQKH :  34
              m                                                                           c        c         k            c  c   q
```

```
                 *       140         *       160         *       180         *       200         *       220         *       240
SexeCSP1  : LVAKTIQAIKDKLPAEYEALIKKHDPENKHQGKL-QEFLQKYSH------------------------------------------------------------------------------ : 126
SexeCSP2  : -------------------------------------------------------------------------------------------------------------------------- :   -
SexeCSP3  : LIAKTIKAIKDKLPSEYEALIKKHDPENKHHDDL-DKFLQKYSH------------------------------------------------------------------------------ : 126
SexeCSP4  : LIRVVANGFQEKTPALWKQLVQKEDPHGQYKETF-TRFINARD------------------------------------------------------------------------------- : 123
SexeCSP5  : GADRVCHYLIDNKPEDWTKLEEKYKSDGSYRIKYLASKLTKDEKELNATKSSEDTNNVSKE------------------------------------------------------------- : 148
SexeCSP6  : QIRRTLAFVQRNYPWEWAKIVRQYG------------------------------------------------------------------------------------------------- : 107
SexeCSP7  : GTRKVLTHLINHEPEMWNQLCEKYDAEGKYRKMY-EDEYKSVKH------------------------------------------------------------------------------ : 122
SexeCSP8  : NVRKTVKALSEKRPDEFAQFRAKFDPKSEYEKDF-MAFVIGTD------------------------------------------------------------------------------- : 122
SexeCSP9  : GAAKVIEHLIKNELDIWHELTDKFDPTGKWRKTY-EDRAKAKGIHIPE-------------------------------------------------------------------------- :  65
SexeCSP10 : -------------------------------------------------------------------------------------------------------------------------- :   -
SexeCSP11 : MGKTLA-------------------------------------------------------------------------------------------------------------------- :  86
SexeCSP12 : TAVKVIKRLKNEYPDEWSKLASRWDPTGDFTRYF-EEFLAKEHYNSIPGSGSALPTSSPLAPPRVPPSPPATTTPTPGPTESTPPRPLILNRFGDDGELMMGSPSSAGITPRPMTQATTKPS : 209
SexeCSP13 : GAYKVIEHLIQNELDTWHELTDKYDSSGKWRKTY-EDRAKANGIIIPE-------------------------------------------------------------------------- :  81
SexeCSP14 : KYDHVLKLLKDNYASFFNEFMQKTAAKKEKH------------------------------------------------------------------------------------------- : 113
SexeCSP15 : IFHRFLLGLKQKLPADYEAFKKKFDPEGQHFHAL-EANVSNS-------------------------------------------------------------------------------- : 123
SexeCSP16 : QIQ----------------------------------------------------------------------------------------------------------------------- :  84
SexeCSP17 : -------------------------------------------------------------------------------------------------------------------------- :   -
SexeCSP18 : LVRLFLEAYSKKSPQEYEKFKDLFDPERK--------------------------------------------------------------------------------------------- : 110
SexeCSP19 : GTRRVLGHLINEEVEYWNRLKAKYDPQSKYTLKY-EQDLRKLKA------------------------------------------------------------------------------ : 128
SexeCSP20 : IVRKLLLGIRAKSEPRFLELLDKYNPDRSNRDAL-YAFLVTGA------------------------------------------------------------------------------- : 122
SexeCSP21 : LIRVVVQGFQNKTPGLWQDLVHKQDPNGQYKEIF-TRFLNGRD------------------------------------------------------------------------------- : 123
SexeCSP22 : LIRVVVQGFQNKTPGLWQDLVHKQDPNGQYKEIF-TRFLNARD------------------------------------------------------------------------------- : 123
SexeCSP23 : LARLFLEAYSKKMPQEYEKFKDLFDPERKYFPKF-EASVAGF-------------------------------------------------------------------------------- : 122
SexeCSP24 : EAGKVIASMMAHDPVAWKVFLTRYDGIKKVQRILG--------------------------------------------------------------------------------------- : 153
SexeCSP25 : SSDKVIRHLVNKRPDLWQELSGKYDPENIYQERY-KTQLDAVKRH----------------------------------------------------------------------------- : 127
SexeCSP26 : GTRKVIKHLINHEPEFWKQLCDKYDPQRKYTTAY-EEELKTL-------------------------------------------------------------------------------- : 124
SexeCSP27 : KYDRVLKLLHDDYETFYNELIKKVASEKE--------------------------------------------------------------------------------------------- : 114
SexeCSP28 : IFHKFLLGLKQKLPSDYDAFKNKYDPEGIHFHSL-EAAVAKF-------------------------------------------------------------------------------- : 123
SexeCSP29 : IFHRFLLGLKQKLPADYEAFKKKFDPEGLHFHTL-EANVANS-------------------------------------------------------------------------------- :  75
```

```
                   *        260         *        280         *        300         *        320
SexeCSP1  : ------------------------------------------------------------------------------- :   -
SexeCSP2  : ------------------------------------------------------------------------------- :   -
SexeCSP3  : ------------------------------------------------------------------------------- :   -
SexeCSP4  : ------------------------------------------------------------------------------- :   -
SexeCSP5  : ------------------------------------------------------------------------------- :   -
SexeCSP6  : ------------------------------------------------------------------------------- :   -
SexeCSP7  : ------------------------------------------------------------------------------- :   -
SexeCSP8  : ------------------------------------------------------------------------------- :   -
SexeCSP9  : ------------------------------------------------------------------------------- :   -
SexeCSP10 : ------------------------------------------------------------------------------- :   -
SexeCSP11 : ------------------------------------------------------------------------------- :   -
SexeCSP12 : TTMKTPSTRPVPPRPTMMTWAGAASNTQATRFPLRPVSDISPPYSTAITLIDQIGYKIIKTTELVTDILRNTVRAVVGR : 288
SexeCSP13 : ------------------------------------------------------------------------------- :   -
SexeCSP14 : ------------------------------------------------------------------------------- :   -
SexeCSP15 : ------------------------------------------------------------------------------- :   -
SexeCSP16 : ------------------------------------------------------------------------------- :   -
SexeCSP17 : ------------------------------------------------------------------------------- :   -
SexeCSP18 : ------------------------------------------------------------------------------- :   -
SexeCSP19 : ------------------------------------------------------------------------------- :   -
SexeCSP20 : ------------------------------------------------------------------------------- :   -
SexeCSP21 : ------------------------------------------------------------------------------- :   -
SexeCSP22 : ------------------------------------------------------------------------------- :   -
SexeCSP23 : ------------------------------------------------------------------------------- :   -
SexeCSP24 : ------------------------------------------------------------------------------- :   -
SexeCSP25 : ------------------------------------------------------------------------------- :   -
SexeCSP26 : ------------------------------------------------------------------------------- :   -
SexeCSP27 : ------------------------------------------------------------------------------- :   -
SexeCSP28 : ------------------------------------------------------------------------------- :   -
SexeCSP29 : ------------------------------------------------------------------------------- :   -
```
