# Supplementary material for: Sex- and stage-dependent expression patterns of odorant-binding and chemosensory protein genes in Spodoptera exempta
Source: PeerJ. 2021 Sep 13;9:e12132. doi: 10.7717/peerj.12132 (PMC8445084; doi:10.7717/peerj.12132)
Supplement: Supplemental Information 5 [file peerj-09-12132-s005.doc]

**Table S2.** Primers used in this study.

| Primer name | Primer sequence (5'-3') | Instruction | PCR program |
| --- | --- | --- | --- |
| SexeOBP1 F | ATGAATAATTTATTTGTGGTGT | Amplification and sequencing of *odorant-binding protein* genes in *Spodoptera exempta* | 30 s at 94 ^o^C, 30 s at 55 ^o^C, and 45 s at 72 ^o^C for 40 cycles |
| SexeOBP1 R | TGTTCGGGTCTTTTTCATAC |  |  |
| SexeOBP2 F | ATGCAGAAGGAAACGAATATG |  |  |
| SexeOBP2 R | GGAAAGTAAAATATACCAGGAT |  |  |
| SexeOBP3 F | ATGAAACTGTTTCTATTATTCTGT |  |  |
| SexeOBP3 R | ACGCCATCTTCGAAACATT |  |  |
| SexeOBP4 F | CTAATTATGAAAACGTGTACC |  |  |
| SexeOBP4 R | AACCGTTTTATTTTAAACTTTGC |  |  |
| SexeOBP5 F | GAACAGTCCCAAAGATCAAA |  |  |
| SexeOBP5 R | CATTTCACATGTTCGTAGAG |  |  |
| SexeOBP6 F | ATGTCGTTCTCAAGTATAATTTG |  |  |
| SexeOBP6 R | CTAGAATCATCTAGAAAGATATC |  |  |
| SexeOBP7 F | GAGGTTAAGTGAAAATGATCT |  |  |
| SexeOBP7 R | ATGTGGAGCTAAATTATTAGAC |  |  |
| SexeOBP8 F | TTAAGTAAGGAACACCATGC |  |  |
| SexeOBP8 R | GTTTCGTTGGCAGACAT |  |  |
| SexeOBP9 F | AGTGAAATCATATCAAAATGACC |  |  |
| SexeOBP9 R | TTTCACATTAGTCGCAAAAG |  |  |
| SexeOBP11 F | ATGGATCGGAAAAGAACATGT |  |  |
| SexeOBP11 R | TGCGAAAATAAAATCTTCTGG |  |  |
| SexeOBP12 F | ATGTTAAGAGTGGGAGTGGCAG |  |  |
| SexeOBP12 R | GATACAGGGAATTTCGATCTAG |  |  |
| SexeOBP13 F | AGTGCAAAGCCAAAGAAAT |  |  |
| SexeOBP13 R | AAAACAGAAACAATTCGTGC |  |  |
| SexeOBP14 F | ATGTCTAAACTTTCTTGTATAGTCG |  |  |
| SexeOBP14 R | GATGATAGCAGTTTTATGTTCC |  |  |
| SexeOBP15 F | ATGTACAAGTTTGCTTGTTTGAT |  |  |
| SexeOBP15 R | ATTGATACTTACAACTTCAGCTTTC |  |  |
| SexeOBP16 F | ATGACTTTTGAAGGGCAAATAG |  |  |
| SexeOBP16 R | TGTAAGAGTTCGTCACCACGAC |  |  |
| SexeOBP17 F | ATGAGTACCTGTACTGATTTGACTC |  |  |
| SexeOBP17 R | GAAATCAAAATCGAAGCCATATT |  |  |
| SexeOBP18 F | ATGAAGACTCTGTTGGTTTTCG |  |  |
| SexeOBP18 R | AAATCGCATGCTTGGGGTCT |  |  |
| SexeOBP20 F | AGGAGGACGGGTAATATAAG |  |  |
| SexeOBP20 R | GTAACACAATCATCCCTGG |  |  |
| SexeOBP21 F | ATGTTCCACTCTGGTGTTGTGT |  |  |
| SexeOBP21 R | CTCGAGGTCAAATCCGAGTTGT |  |  |
| SexeOBP22 F | ATGTACTCCATCAACTATTTTCTT |  |  |
| SexeOBP22 R | AACATAAAATTAGCAGAGTCGA |  |  |
| SexeOBP24 F | ATGGCTAAAGTTATTTACATAGTC |  |  |
| SexeOBP24 R | GCAGAATCGCTAAACTTATTTG |  |  |
| SexeOBP26 F | ATGTCAGTTATGAGACGTTCAT |  |  |
| SexeOBP26 R | CCACATTCGTCTCCGACT |  |  |
| SexeOBP27 F | ATGTGGAATTGTTTAGTAGTGTT |  |  |
| SexeOBP27 R | CTTGAATCCAAACTTGGGAG |  |  |
| SexeOBP29 F | CTTTCAGAAATACTCATCTTCAC |  |  |
| SexeOBP29 R | GTCGGGGTCTCTGTAGGACAC |  |  |
| SexeOBP30 F | AGTGGTCTTGCAAGCGCTG |  |  |
| SexeOBP30 R | GAGTTGTTGTCCGACTCGCT |  |  |
| SexeOBP31 F | ATGAAGTATAAAGCAAGGCG |  |  |
| SexeOBP31 R | ATTCATCCTTGACCAACTTC |  |  |
| SexeOBP35 F | CAAAAAGCGGTTTCTAACAA |  |  |
| SexeOBP35 R | GAGGATTAAAAGGAATGGGG |  |  |
| SexeOBP36 F | TGCTGCTAATAAAAGTATTCAG |  |  |
| SexeOBP36 R | TGGCATTGATCTTACTTCTTGT |  |  |
| SexeOBP37 F | ATTCTAATCCCAGCCATTCTC |  |  |
| SexeOBP37 R | GCAAATAAAACGTGTGATTTAG |  |  |
| SexeOBP38 F1 | AATTGTAGTTGTTGGAGAGC |  |  |
| SexeOBP38 R1 | AACTACAGTCAAAGAACGTA |  |  |
| SexeOBP40 F | GTTTATAAGAAAGCTGAAGTTATC |  |  |
| SexeOBP40 R | GGGATCTCGCTCTTGTGTT |  |  |
| SexeCSP2 F | ATGAATTTCCTCGTGTTATCAATC | Amplification and sequencing of *odorant-binding protein* genes in *Spodoptera exempta* | 30 s at 94 ^o^C, 30 s at 55 ^o^C, and 45 s at 72 ^o^C for 40 cycles |
| SexeCSP2 R | TTCAAGCAACACTTTATCTTACCT |  |  |
| SexeCSP4 F | ATGAAGGGCATCCTAGTTCTATG |  |  |
| SexeCSP4 R | CTGGCGTTTATGAATCTGGTG |  |  |
| SexeCSP5 F | ATGATGACGAAATCATTTATATC |  |  |
| SexeCSP5 R | GAAACATTATTTGTATCTTCACTGG |  |  |
| SexeCSP6 F | ATGCAGGTCATAGTAGTTTTAGTCG |  |  |
| SexeCSP6 R | CATATTGGCGGACGATTTTG |  |  |
| SexeCSP8 F | ATGAACGCTCTCCTGATAGCTG |  |  |
| SexeCSP8 R | AGTACCGATGACGAAAGCCAT |  |  |
| SexeCSP9 F | ATGGTGGACGCCATTGAGAC |  |  |
| SexeCSP9 R | CTGGGATGTGGATGCCCTTG |  |  |
| SexeCSP10 F1 | CCCGACGACTCCCACTACA |  |  |
| SexeCSP10 R1 | GATGCACTTGACGTAGGGC |  |  |
| SexeCSP11 F | ATGAAGGTAGCACTACTCACACTC |  |  |
| SexeCSP11 R | GCCAAAGTCTTACCCATTTGCT |  |  |
| SexeCSP12 F | ATGCGTGTAGTGTTGTTTATTTG |  |  |
| SexeCSP12 R | GACAGCTCTCACTGTGTTTCTA |  |  |
| SexeCSP13 F1 | AGAGGAAAGTGCAGTCCTGAG |  |  |
| SexeCSP13 R1 | TCTTCGTATGTCTTCCTCCACT |  |  |
| SexeCSP16 F | ATGCAGATCAAATACGCGTTG |  |  |
| SexeCSP16 R | GTATTTGCTTGGTTTCTTGTG |  |  |
| SexeCSP17 F | ATGAGGGTTCTAGTTGCTTTG |  |  |
| SexeCSP17 R | GTATGCCTTGAGCAGTCTGTC |  |  |
| SexeCSP18 F | TGGTGAAGGCTCAAGAAACGT |  |  |
| SexeCSP18 R | GTGGGCTTTTCTTCGAGTAGG |  |  |
| SexeCSP19 F2 | ATGAAATCCTTCATAGTTGTGT |  |  |
| SexeCSP19 R2 | CTTTTAGTTTTCTAAGGTCCTG |  |  |
| SexeCSP20 F | ATGAGGAGCTGGCTGTTATG |  |  |
| SexeCSP20 R | CGGTGACAAGGAAAGCGT |  |  |
| SexeCSP22 F | ATGTTCGGCTGCTGACTG |  |  |
| SexeCSP22 R | CCTGGGGTCTTGTTTTGG |  |  |
| SexeCSP23 F | ATGAAGCACTTTTTAGTTCTCTT |  |  |
| SexeCSP23 R | CAGCAACTGAAGCTTCGAACT |  |  |
| SexeCSP24 F | ATGTGGGTTCAGGCTATCCC |  |  |
| SexeCSP24 R | TCTTTGAACTTTTTTGATTCCG |  |  |
| SexeCSP25 F1 | CTTATCATCGCCGTCGTTTTAT |  |  |
| SexeCSP25 R1 | GCATCAAGCTGGGTTTTGTA |  |  |
| SexeCSP26 F | TCTTTACGAAGGCTAACTTG |  |  |
| SexeCSP26 R | AACAGTCATCATCAACCAAA |  |  |
| SexeCSP27 F | AAGGTACTAGTAGTGTTGTCCG |  |  |
| SexeCSP27 R | CTCCTTCTCGGAAGCTAC |  |  |
| SexeCSP28 F | ATGAAGTGTATCTACGTGTTGTCG |  |  |
| SexeCSP28 R | GTGTTCTCGCTGAGGACAAGTA |  |  |
| SexeCSP29 F6 | CACGCTGTTGCTGCCGATTT |  |  |
| SexeCSP29 R6 | TGGCCTCAAGAGTATGGAAGTG |  |  |
| SE-CSP30-F | ATTGAACCAGGTCAAGTTTC |  |  |
| SE-CSP30-R | GTCTCTCAAACAAAATCCCT |  |  |
| SE-CSP32-F | GAGATTTTTGATCTGAAGCTAAG |  |  |
| SE-CSP32-R | GAACTGAAATCTCACATCAGG |  |  |
| SE-CSP33-F | TCTAAATGATATCGCACTGTA |  |  |
| SE-CSP33-R | ATTATTGGCTCTAAGCAGTG |  |  |
| SE-OBP9-qF | TGGGATTGTGTCAGAAGATGGTATGC | Detection of DEGs from larvae of *S.exempta* with qPCR | 45 cycles of 95°C for 15 s, 60 °C for 34 s |
| SE-OBP9-qR | GGGCTTTCCATTCCGTTCTCCAG |  |  |
| SE-OBP13-qF | CATTGTCTTCTGTATTGTGGCTGCTAG |  |  |
| SE-OBP13-qR | GCCTCGCTCACTCCATGTTCTTC |  |  |
| SE-OBP15-qF | CCACACATACAGGAGTGCTCAAAGG |  |  |
| SE-OBP15-qR | CGCAGCTTAGGAAACAAGAGTTGATG |  |  |
| SE-OBP16-qF | CGCCGTTTATGCAGAGGAGGATG |  |  |
| SE-OBP16-qR | CGAGAGCACCCACATTACCTGATC |  |  |
| SE-OBP18-qF | CCTGACAGATGAGCAGAAGGAGAAAC |  |  |
| SE-OBP18-qR | CACCACCCTTGAGCTTGTTGACTAG |  |  |
| SE-OBP26-qF | GCGTTCATGCTCAATCTGCTGATG |  |  |
| SE-OBP26-qR | TCCAAATTCTTCGGCACACTCTAGTAC |  |  |
| SE-OBP29-qF | ATCGGATGTCGGAGAGACCTGTG |  |  |
| SE-OBP29-qR | TGCAATCGTTAAACACCTTCGCTTTC |  |  |
| SE-OBP30-qF | CGACCAGATCCTCACATGACTTGC |  |  |
| SE-OBP30-qR | ACATCGCCTTCTCCTCCTTACCC |  |  |
| SE-OBP31-qF | GCTTCGTCGCCTGCATATTGAAAC |  |  |
| SE-OBP31-qR | AGCACTCGCTCACAGCAATCTTC |  |  |
| SE-OBP35-qF | CCGGATTGGTCGTATGCGAATTTTC |  |  |
| SE-OBP35-qR | TTAGGAGAGAGCCGCTTCACAGG |  |  |
| SE-OBP37-qF | ATTCTAATCCCAGCCATTCTCTTCCAG |  |  |
| SE-OBP37-qR | TTTCTTAGCTTCGCCTACAACCTCAG |  |  |
| SE-CSP2-qF | ACGGATAGATACGACCACATCAACATC |  |  |
| SE-CSP2-qR | GTTCTCTGCCTTCAGGAGTACATCTTC |  |  |
| SE-CSP4-qF | ACCGAGGCAGCGTCATTTAATCC |  |  |
| SE-CSP4-qR | TCTGGCGTTTATGAATCTGGTGAAGG |  |  |
| SE-CSP5-qF | ACGGAATCATCCACATACACGACTAAG |  |  |
| SE-CSP5-qR | GGTCCAAGATCCATCAGGCAGTTG |  |  |
| SE-CSP8-qF | CTGCTCCGTCGAACATTCTGCTG |  |  |
| SE-CSP8-qR | GCGAACTCATCAGGTCTCTTCTCAC |  |  |
| SE-CSP9-qF | AGCTGCCAAGGTCATCGAACATC |  |  |
| SE-CSP9-qR | CGGTCCTCGTAAGTCTTCCTCCAC |  |  |
| SE-CSP10-qF | ATGAAGTCCATGATCGTGCTCTGC |  |  |
| SE-CSP10-qR | TTGTCGTAGCGGTCGGTGTAGTG |  |  |
| SE-CSP12-qF | CCACCGAGACCACTTATACTGAACAG |  |  |
| SE-CSP12-qR | TTGGAGTAATTCCTGCTGACGATGG |  |  |
| SE-CSP13-qF | AGTGCAGTCCTGAGGGCAAG |  |  |
| SE-CSP13-qR | CCAGGTGTCCAGCTCGTTCT |  |  |
| SE-CSP17-qF | GCTTTGTCGTGCTTGGTCGT |  |  |
| SE-CSP17-qR | GCCTTGAGCAGTCTGTCGTTG |  |  |
| SE-CSP19-qF | TCAAGCACTTACACTGACAGATACGAC |  |  |
| SE-CSP19-qR | AGCCTCCCTGATGTGAGATTTCAATTC |  |  |
| SE-CSP23-qF | GCAGTATGACAATGTAAACGGAGAAGC |  |  |
| SE-CSP23-qR | AGCGTCGCCCCTAAAACAATCG |  |  |
| SE-CSP25-qF | AACTCTTCCTGACGCCTTAGAACATG |  |  |
| SE-CSP25-qR | CCTGATAACTCCTGCCACAAGTCTG |  |  |
| SE-CSP26-qF | TTCCCTATGTTAAGTGCTGCCTTGAC |  |  |
| SE-CSP26-qR | TTGATGACCTTGCGAGTACCATTCTG |  |  |
| SE-CSP28-qF | AGGACAAGTACAGCACAGAGAATGATG |  |  |
| SE-CSP28-qR | CCACAGCGTGACAGGTGATAGAATC |  |  |
| SE-CSP30-qF | CTGGCGAGATGAGCTTCTTCTTAGAC |  |  |
| SE-CSP30-qR | ATATTTTGTACGAGGCCCACCAAGAC |  |  |
| SEACTINF | CCGCTGAGAGGGAAATCG |  |  |
| SEACTINR | TACCCAAGAAGGAAGGCT |  |  |
| SEGAPDHF | TTTCCGTGTTCCTGTTGC |  |  |
| SEGAPDHR | CTTGAGGGGTCCTTGGGC |  |  |
